# Advances in adoptive cellular immunotherapy and therapeutic breakthroughs in multiple myeloma

**DOI:** 10.1186/s40164-024-00576-6

**Published:** 2024-10-28

**Authors:** Jingjing Pu, Ting Liu, Amit Sharma, Liping Jiang, Feng Wei, Xiubao Ren, Ingo G. H. Schmidt-Wolf, Jian Hou

**Affiliations:** 1 Department of Integrated Oncology, Center for Integrated Oncology (CIO) Bonn, University Hospital Bonn, 53127 Bonn, NRW Germany; 2 Translational Biogerontology Lab, German Center for Neurodegenerative Diseases (DZNE), 53127 Bonn, NRW Germany; 3 Wuxi Maternity and Child Health Care Hospital, Wuxi School of Medicine, Jiangnan University, Wuxi, 214002 Jiangsu China; 4 Tianjin Medical University Cancer Institute and Hospital, National Clinical Research Center for Cancer, Tianjin, 300070 China; 5 Renji Hospital, Shanghai Jiao Tong University School of Medicine, Shanghai, 200127 China

**Keywords:** Multiple myeloma, Cell therapy, Immunotherapy, CAR-T, CAR-NK, CIK

## Abstract

The basic idea of modulating the immune system to better recognize and fight tumor cells has led to the successful introduction of adoptive cellular immunotherapy (ACT). ACT-based treatment regimens, in which the patient's own immune cells are isolated and subsequently expanded (ex vivo) and reinfused, have also contributed significantly to the development of a personalized treatment strategy. Complementing this, the unprecedented advances in ACTs as chimeric antigen receptor (CAR)-T cell therapies and their derivatives such as CAR-NK, CAR-macrophages, CAR-γδT and CAR-NKT have further maximized the therapeutic outcomes. Herein, we provide a comprehensive overview of the development of ACTs in multiple myeloma (MM) and outline how they have evolved from an experimental form to a mainstay of standard clinical settings. Besides, we provide insights into cytokine-induced killer cell (CIK) therapy, an alternative form of ACT that (as CIK or CAR-CIK) has enormous potential in the clinical spectrum of MM. We also summarize the results of the major preclinical and clinical studies of adoptive cell therapy in MM and address the current challenges (such as cytokine release syndrome (CRS) and neurotoxicity) that limit its complete success in the cancer landscape.

**Supplementary Information:**

The online version contains supplementary material available at 10.1186/s40164-024-00576-6.

## Introduction

**Fig. 1:**
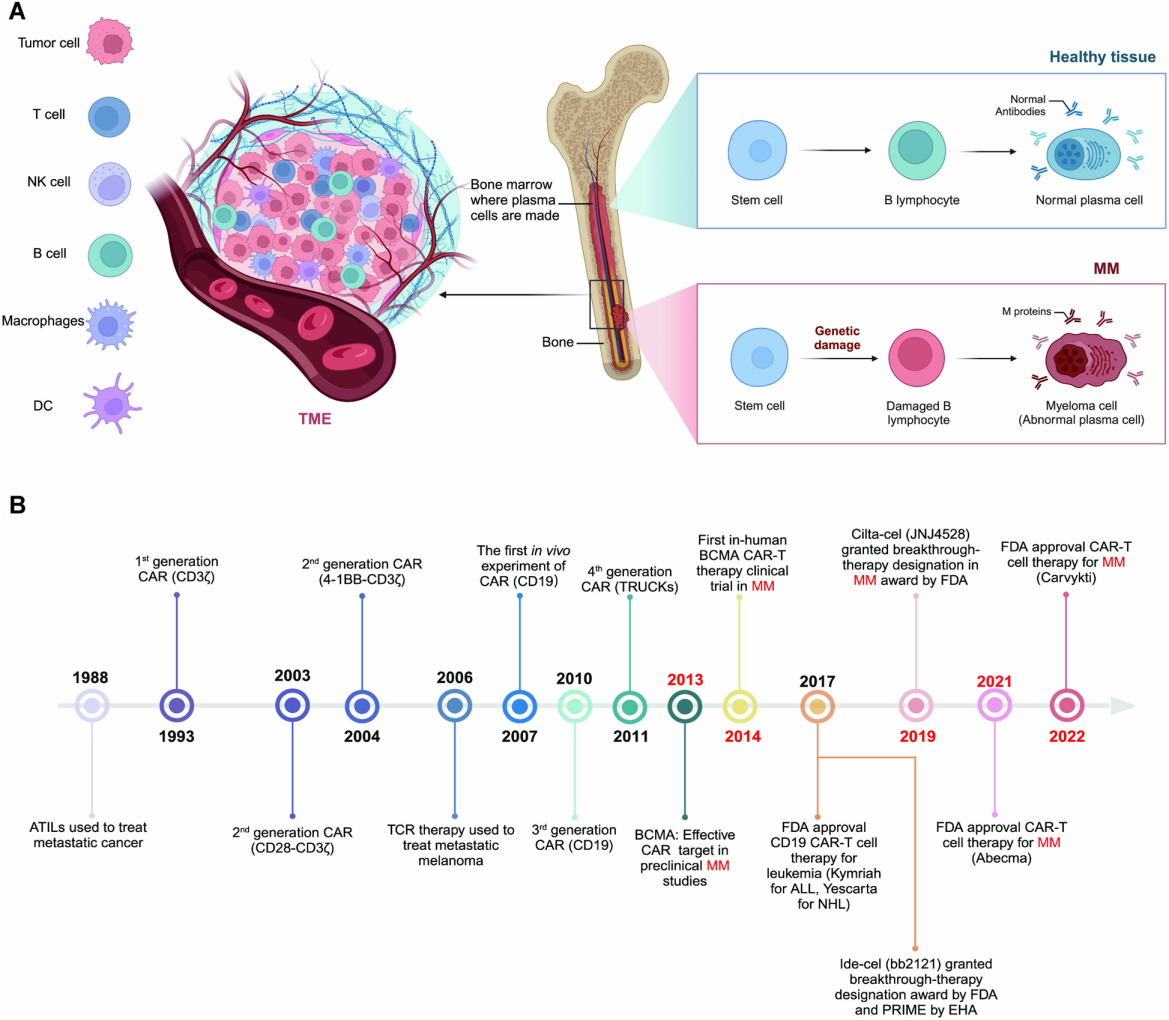
**A** The process of normal immune cell development versus the development of MM. In healthy bone marrow, stem cells develop into B lymphocytes which then mature into plasma cells, producing normal antibodies. In contrast, in multiple myeloma, genetic damage to stem cells leads to the formation of abnormal B lymphocytes, which evolve into myeloma cells. These cancerous cells produce an abnormal protein known as M protein, disrupting normal blood cell production and immune functions. Immunosuppressive Tumor Microenvironment (TME): The TME consists of tumor cells, stroma, and various immune cells, such as dendritic cells (DCs), T cells, NK cells, B cells, and macrophages, which often become exhausted and contribute to the immunosuppressive nature of the environment. **B** Timeline of significant milestones in CAR-T cell therapy development. Beginning with the use of adoptive transfer of tumor-infiltrating lymphocytes (ATILs) for metastatic cancer in 1993, significant milestones include the introduction of various generations of CAR constructs, such as CD3ζ (first generation) and 4-1BB-CD3ζ (second generation). Noteworthy is the FDA's approval of the first CAR-T cell therapies: Kymriah in 2017 for ALL and Yescarta for NHL, followed by approvals for multiple myeloma treatments, Abecma in 2021, and Carvykti in 2022. Additionally, the figure notes the ide-cel (bb2121) receiving breakthrough therapy designation, highlighting the ongoing innovation and regulatory endorsement in the CAR-T field. Each milestone in this timeline underscores the rapid evolution and increasing complexity of CAR-T cell therapies, showcasing both clinical and regulatory advancements that have significantly impacted cancer treatment paradigms. Figure created with BioRender.com

Multiple myeloma (MM) is a malignancy of plasma cells in the bone marrow, comprising around 10% of all hematologic cancers. This disorder arises from the genetic alteration of stem cells, which causes normal B lymphocytes to morph into malignant myeloma cells. These cells produce dysfunctional M proteins, which contribute to disease progression and associated symptoms such as severe bone damage, kidney dysfunction, anemia, and elevated calcium levels [1–3] (Fig. 1A). In the U.S., MM predominantly affects older adults, typically beginning around age 69, with a prevalence rate of 7 per 100,000 people annually [4]. It often develops from conditions like monoclonal gammopathy of undetermined significance (MGUS) or smoldering MM, found in 3% of those over 50 [5, 6]. MM is notably more prevalent among individuals of African descent and in industrialized areas such as the U.S., where it constitutes 1.8% of all cancer cases [7]. In 2022, there were about 34,470 new cases and 12,640 deaths due to MM in the U.S., and men are 1.5 times more likely to be affected than women. Over the past two decades, the treatment landscape for MM has been transformed through the widespread adoption of autologous stem cell transplantation (ASCT) and the approval of innovative medications and strategies. A variety of new drugs, including histone deacetylase inhibitors, proteasome inhibitors, immunomodulatory drugs, monoclonal antibodies, and other targeted therapies, have been developed. These advances have not only improved the five-year survival rate but also shifted treatment approaches towards more intricate combinations, such as triple therapy, and extended treatment durations to enhance patient outcomes. Notably, the tumor microenvironment (TME) in MM plays a crucial role in disease pathogenesis, progression, and therapeutic resistance [8, 9] (Fig. 1A). Targeting the TME offers a promising strategy to enhance treatment outcomes for MM patients. Current research aims to understand the complexities of the TME and develop new therapies to exploit its weaknesses [10]. Despite advances in these areas, MM remains incurable, with current treatments often limited by resistance and relapse. This highlights the urgent need for innovative therapeutic approaches to achieve a cure [11–16].

Over recent decades, advancements in adoptive cellular immunotherapy (ACT) have not only revolutionized the therapeutic landscape but have also progressively redefined the paradigms of clinical care for MM [17]. This review delves into the transformative journey of cell therapy in MM, tracing its origins from an experimental stratagem to its status as a cornerstone in the management of this challenging disease. Initially, the adoption of ASCT marked a significant breakthrough, enhancing survival rates and setting a new benchmark for care [18–20]. The introduction of ASCT in the late twentieth century heralded the first wave of innovations that provided a glimmer of hope against a once grim prognosis [21–23]. Building on this foundation, the emergence of chimeric antigen receptor (CAR)-T cell therapies catalyzed a seismic shift in the treatment modalities available for MM [24, 25] (Fig. 1B). Particularly, the development of CAR-T cells targeting the B cell maturation antigen (BCMA) has demonstrated remarkable efficacy in treating patients with relapsed/refractory multiple myeloma (RRMM), offering unprecedented response rates and opening new avenues for remission [26–28].

Further, this review explores the burgeoning role of alternative cell-based therapies, such as natural killer (NK) cells and T cell receptor (TCR) engineered cells [29–32]. These therapies are not merely adjuncts to existing treatments but are pivotal in addressing ongoing challenges such as antigen escape and resistance to CAR-T cell therapy [28, 33]. Moreover, we address the critical management of adverse effects, including cytokine release syndrome (CRS) and neurotoxicity [34–36], which are significant considerations in the deployment of these potent therapeutic options.

**Fig. 2:**
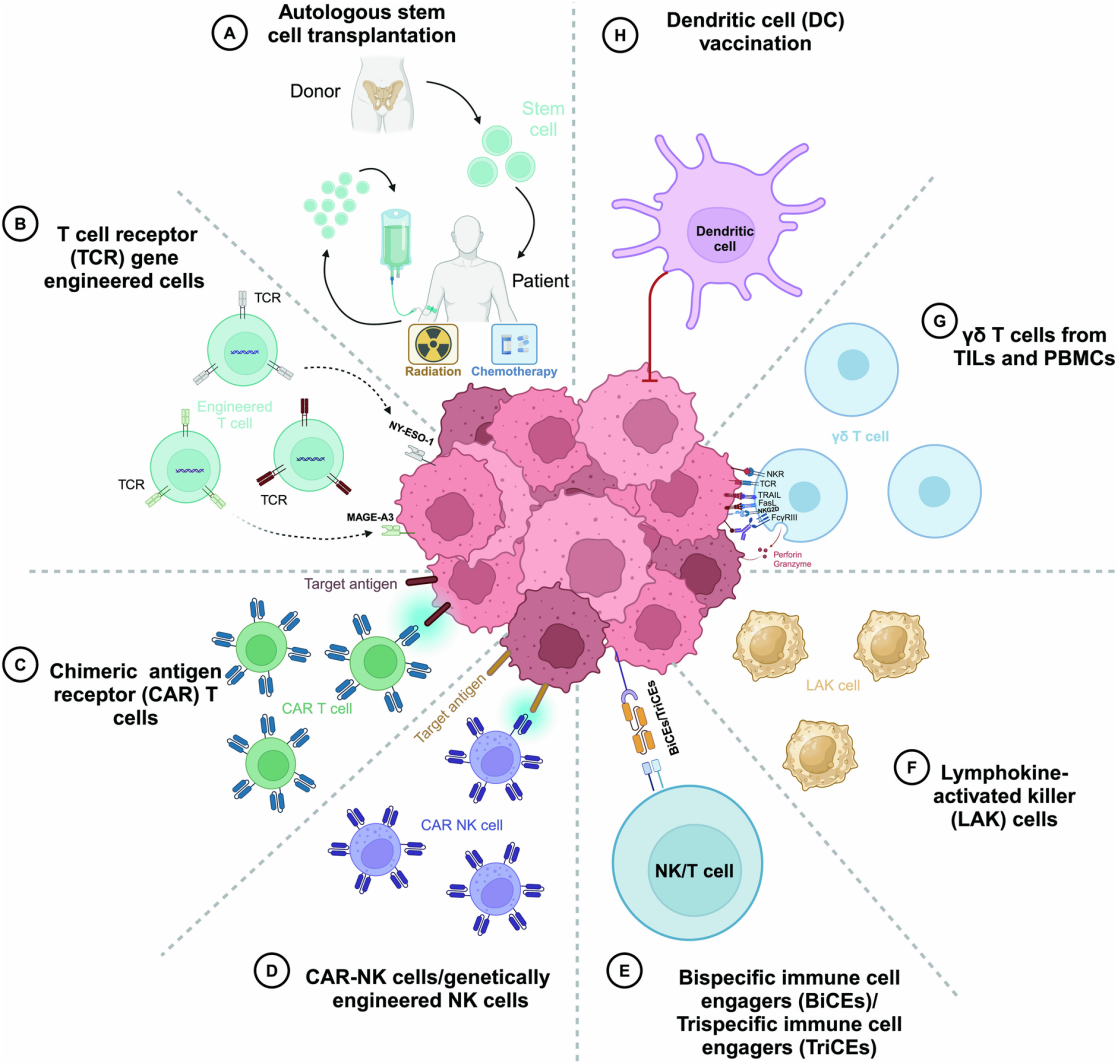
Cell therapies for multiple myeloma (MM). **A** Autologous stem cell transplantation, remains the cornerstone of treatment for MM; **B** T cell receptor (TCR) gene engineered cells enhance a patient’s T cells by incorporating a receptor designed to target specific antigens, such as NY-ESO-1, and MAGE-A3, present on myeloma cells. These targeted antigens, derived from proteins commonly found in cancer cells, enable the modified T cells to recognize and destroy tumor cells that exhibit these antigens once reintroduced into the patient; **C** Chimeric antigen receptor (CAR) T cells, a groundbreaking advancement in MM, designed to enhance the body’s immune response against malignant cells; **D** CAR-NK cells/genetically engineered NK cells, which express engineered receptors targeting one or more antigens, facilitate the activation of immune cells against MM cells; **E** Bispecific immune cell engagers (BiCEs)/Trispecific immune cell engagers (TriCEs) are a form of immunotherapy that targets cancer cells. By binding to both immune cells (NK cells or T cells) and MM cells, BiCEs or TriCEs bring these cells into close contact, enabling NK or T cells to effectively kill the MM cells; **F** Lymphokine-activated killer (LAK) cells, are a promising immunotherapy for MM, but further research and clinical trials are needed to fully explore and optimize their therapeutic potential; **G** γδ T cells from tumor-infiltrating lymphocytes (TILs) and peripheral blood mononuclear cells (PBMCs), a subset of T cells with non-MHC‐restricted cytotoxic activity, are notable for their ability to directly kill MM cells and modulate the immune response. This dual function can promote tumor eradication or facilitate tumor immune evasion; **H** Dendritic cell (DC) vaccination involves using autologous dendritic cells that have been loaded with peptides or tumor-derived proteins to activate cytotoxic T cell responses in MM patients. Figure created with BioRender.com

As we stand on the brink of significant advancements, this review anticipates future innovations, including combination therapies and genetically tailored approaches designed to improve efficacy, safety, and personalized treatment outcomes. By integrating historical achievements with current research directions, we endeavor to shed light on the path toward achieving durable remissions and, ultimately, a cure for MM or RRMM. This review outlines key historical milestones and emphasizes the transformative potential of cell therapy in MM. The evolution of cell therapies, illustrated in Fig. 2 and elaborated in the text, shows great promise for MM treatment. Additionally, Table 1 and Supplementary Table 1 summarize the results from major pre-clinical and clinical adoptive cell therapy studies for MM.

**Table 1:** Clinical studies of adoptive cell therapies for MM treatment

Type	Phase	Clinical design	Clinical response	Most common grade 3/4/5 toxicities	References
TCR gene engineered T cells	I/II (NCT01352286)	Treatment: Received NY-ESO-1-specific TCR-engineered T cells after ASCT Protocol: Twenty patients with antigen-positive MM received an average 2.4 × 10^9^ NY-ESO-1-LAGE-1 TCR-engineered T cells for 2 days after ASCT	CR: 80%; PFS: 19.1 months	GVHD (17.4%), rash (6%), hypotension (6%)	[37]
CAR-T cells	I (NCT02658929)	Treatment: BCMA-targeted CAR-T cell (Idecabtagene vicleucel; Bb2121) Protocol: CTX + FAMP + 150 or 450 × 10^6^ cells/pt	ORR: 75.8%; CR: 38.7%; mPFS: 18.1 months	Neutropenia (88.7%), anemia (56.5%), infection (22.6%), CRS (6.5%), thrombocytopenia (56.5%), leukopenia (61.3%), lymphopenia (35.5%), ICANS (1.6%), second primary malignancy (3.2%)	[38]
	I/II (NCT03090659)	Treatment: BCMA-targeted CAR-T cell (LCAR-B38M) Protocol: CTX + avg 0.5 × 10^6^ cells/kg 3 split infusions	ORR: 87.8%; CR: 73%; MRD negativity: 67.6%; 5-year PFS: 21%; 5-year OS: 49.1%	Neutropenia (85.3%), thrombocytopenia (58.8%), and hepatic disorder (38.3%)	[39]
	Ib/II (NCT03548207)	Treatment: BCMA-targeted CAR-T cell (JNJ-4528) Protocol: CTX + FAMP + avg 0.75 × 10^6^ cells/kg	ORR: 97.9%; sCR: 82.5%; 27-month PFS: 54.9%; 27-month OS: 70.4%	Neutropenia (94.8%), anemia (68%), thrombocytopenia (59.8%), leukopenia (60.8%), lymphopenia (50.5%), metabolism and nutrition disorders (18.5%), CRS (5.1%), ICANS (12.3%)	[40]
	Ib (NCT02546167)	Treatment: BCMA-targeted CAR-T cell (CART-BCMA) Protocol: None or CTX + 1–5 × 10^7^ or 1–5 × 10^8^ cells/pt	ORR: 48%; CR: 8%; VGPR: 20%; PR: 20%	Leukopenia (44%), neutropenia (44%), lymphopenia (36%), CRS (32%), ICANS (12%)	[41]
	I (NCT03070327)	Treatment: BCMA-targeted CAR-T cell (MCARH171) Protocol: CTX + FAMP + 72, 137, 475 or 818 × 10^6^ cells/pt (1 or 2 doses)	ORR:64%	CRS (23%), ICANS (36%)	[42]
	I (NCT03455972)	Treatment: CD19-targeted CAR-T cell and BCMA-targeted CAR-T cell Protocol: anti-CD19 CAR-T cells were infused at a fixed dose of 1.0 × 10^7^/kg body weight on day 0, followed by 2.0 × 10^7^/kg of anti-BCMA CAR-T cells on day 1 and 3.0 × 10^7^/kg of anti-BCMA CAR -T cells on day 2	ORR: 100%; sCR: 90%; CR: 10%	Neutropenia (20%), lymphopenia (100%), thrombocytopenia (70%), anemia (50%), elevated bilirubin (10%), hypotension (10%), respiratory infection (50%)	[43]
	II (ChiCTR17011272)	Treatment: CD19-targeted CAR-T cell and BCMA-targeted CAR-T cell Protocol: CTX + FAMP + humanized anti-CD19 CAR-T cells (1 × 10^6^ cells/kg) and murine anti-BCMA CAR-T cells (1 × 10^6^ cells/kg)	ORR: 95%; sCR: 43%; CR: 14%; VGPR: 24%; PR: 14%	Neutropenia (86%), anaemia (62%), thrombocytopenia (62%), CRS (4%)	[44]
	I/II (ChiCTR2000033567)	Treatment: Bispecific BC19 CAR-T cell targeting BCMA/CD19 Protocol: Not found	ORR: 92%; mPFS: 19.7 months; mOS: 19.7 months	Neutropenia (98%), anemia (64%), thrombocytopenia (66%), leukopenia (96%), AST increased (24%), Pyrexia (24%), CRS (8%)	[45]
	I (ChiCTR1800018143)	Treatment: Bispecific BM38 CAR-T cell targeting BCMA/CD38 Protocol: CTX + FAMP + anti BM38 CAR-T cells (0.5, 1.0, 2.0, 3.0 and 4.0 × 10^6^ cells/kg)	ORR: 87%; CR: 52%	CRS (22%), neutropenia (87%), anemia (13%), thrombocytopenia (47%), leukopenia (83%)	[46]
	I (ChiCTR1900026286)	Treatment: Bispecific BM38 CAR-T cell targeting BCMA/CD38 Protocol: CTX + FAMP + anti BM38 CAR-T cells	ORR: 88%; CR: 81%	Anemia (37.6%), thrombocytopenia (25%), leukopenia (93.8%), CRS (31.3%)	[47]
	I (ChiCTR1800017051)	Treatment: CD38-targeted CAR-T cell and BCMA-targeted CAR-T cell Protocol: CTX + FAMP + humanised anti-BCMA CAR-T cells (2 × 10^6^ cells/kg) and murine anti-CD38 CAR-T cells (2 × 10^6^ cells/kg)	ORR: 90.9%; CR: 54.5%	Fever (36.4%), leukopenia (36.4%), anemia (27.3%), CRS (27.3%)	[48]
	I/IIa (NCT04662099)	Treatment: Bispecific CS1 CAR-T cell targeting CSI/BCMA Protocol: CTX + FAMP + bispecific CS1-BCMA CAR-T cells (0.75 × 10^6^, 1.5 × 10^6^ and 3.0 × 10^6^ cells/kg)	ORR: 81%; sCR: 38%	CRS (6%), pain (19%), infection (31%), leukopenia (100%), neutropenia (94%), lymphopenia (100%), anemia (13%), thrombocytopenia (31%)	[49]
	I (NCT04555551)	Treatment: GPRC5D-targeted CAR-T cell (MCARH109) Protocol: GPRC5D-targeted CAR-T cells (25 × 10^6^ to 450 × 10^6^ cells in total)	ORR: 71%; CR: 35%; MRD negativity: 47%	CRS (5.9%), ICANS (5.9%)	[50]
	I (NCT04674813)	Treatment: GPRC5D-targeted CAR-T cell (BMS-986393) Protocol: GPRC5D-targeted CAR-T cells (25 × 10^6^ to 450 × 10^6^ cells in total)	Patients with assessable efficacy: ORR: 86%; CR: 38%; patients refractory to prior BCMA-directed therapy: ORR: 85%; CR: 46%	Neutropenia (69%), anemia (31%), thrombocytopenia (30%), CRS (4.3%), ICANS (3%)	[51]
	I (NCT05016778)	Treatment: GPRC5D-targeted CAR-T cell (OriCAR-017) Protocol: GPRC5D-targeted CAR-T cells (1 to 6 × 10^6^ CAR-T cells/kg)	ORR: 100%; sCR: 60%	Neutropenia (100%), thrombocytopenia (90%), leukopenia (90%), anemia (70%)	[52]
CAR-NK cells	I/II (NCT03940833)	Treatment: BCMA-specific CAR-NK 92 cell Protocol: Not found	Not found	Not found	Not found
	I (NCT05182073)	Treatment: Allogenic CAR NK cells with BCMA expression (FT576) Protocol: CTX + FAMP + Daratumumab + Bendamustine + FT576	Not found	Not found	Not found
LAK cells	I/II	Treatment: rIL-2 Protocol: 9 × 10^6^ IU/m^2^ twice daily on day 1 and 2, then 0.9 × 10^6^ IU/m^2^ twice daily from day 3 to 56, every 12 weeks until disease progression	ORR: 35.3%	Not found	[53]
	I	Treatment: rIL-2 Protocol: 0.3 to 4.5 × 10^6^ IU/m^2^ daily from day 1 to 5, then 0.3 × 10^6^ IU/m^2^ daily from day 12 to 21	Not found	Most patients exhibited mild to moderate fever, nausea, diarrhea, and/or skin rash	[54]
γδ T cells	II	Treatment: Zoledronate + IL-2 Protocol: IL-2: 2 × 10^6^–8 × 10^6^ IU/day (increased by 25% each time until toxicity was observed); 4 mg of zoledronic acid were administered on day 2 of each cycle	CR: 18%	Treatment was well tolerated without G3 or 4 toxicities	[55]
	I	Treatment: Pamidronate + IL-2 Protocol: Pamidronate on day 1 followed by increasing dose levels of IL-2 from day 3 to day 8 (0.25–3 × 10^6^ IU/m^2^); pamidronate on day 1, followed directly by IL-2 administration from day 1 to day 6	PR: 33%	Thrombosis (10%)	[56]
	I	Treatment: Zoledronate + IL-2 activated allogeneic γδ T cells from healthy donors Protocol: On average, patients received 2.17 × 10⁶/kg (range 0.9–3.48) γδ T cells. All patients received prior lymphopenia-inducing chemotherapy (FAM 20–25 mg/m^2^ day -6 until day -2 and CTX 30–60 mg/kg day -6 and -5) and were treated with 4 mg zoledronate on day 0 and 1.0 × 10⁶ IU/m^2^ IL-2 on day + 1 until day + 6 for the induction of γδ T cell proliferation in vivo	CR: 75% (patients with plasma cell leukaemia)	Neutropenia (100%)	[57]
	I (NCT04688853)	Treatment: TEG002 cells (autologous T cells transduced with a specific γδTCR) Protocol: Not found	Ongoing	Not found	Not found
DC vaccination	II (NCT02728102)	Treatment: DC/MM fusion vaccine + lenalidomide (Revlimid) + GM-CSF Protocol: DC/MM fusion vaccine was given on day 1 of cycles 2, 3, and 4 of lenalidomide maintenance therapy, starting 90–100 days after auto-HSCT, and continued for 2 years. GM-CSF was given daily for a total of 4 days of each cycle (each cycle lasted 28 days)	CR: Vaccine group vs control group (52.9% vs 50%); VGPR: Vaccine group vs control group (85.3% vs 77.8%)	Blood and lymphatic disorders (52.9%), gastrointestinal disease disorders (14.7%), nervous system disorders (16.2%), metabolism and nutrition disorders (10.3%)	[58]

MM: multiple myeloma; ORR: overall response rate; sCR: stringent complete response; MRD: minimal residual disease; CR: complete responses; VGPR: very good partial response; PR: partial response; OS: overall survival; mOS: median overall survival; PFS: progression-free; mPFS: median progression-free; GVHD: Graft Versus Host Disease; CRS: cytokine release syndrome; ICANS: immune effector cell-associated neurotoxicity syndrome; CTX: cyclophosphamide; FAM: fludarabine; pt: per test; data sourced from the clinicaltrials.gov site and the chictr.org.cn site

## Preclinical and clinical applications of adoptive cell therapies for MM

### Autologous stem cell transplantation

Autologous stem cell transplantation (ASCT) remains the cornerstone of treatment for MM, especially in younger patients under 65 years old who are in good health [59]. This treatment follows a multi-phase therapeutic path that includes induction, high-dose melphalan (HDM) with ASCT, consolidation, and maintenance therapy, the combination of high-dose chemotherapy and ASCT provides the maximum therapeutic benefit in eligible MM patients by leveraging the cytotoxic effects of chemotherapy while ensuring recovery of bone marrow function through stem cell reinfusion. While ASCT itself does not directly target myeloma cells, it enables the use of more aggressive chemotherapy, leading to improved long-term outcomes [18, 60]. ASCT achieves high response rates and significantly extends both progression-free (PFS) and overall survival (OS), outperforming standard chemotherapy regimens [18, 61, 62]. Recent guidelines advocate for induction therapy with bortezomib, thalidomide, and dexamethasone (VTd) or bortezomib, lenalidomide, and dexamethasone (VRd) combined with the anti-CD38 monoclonal antibody daratumumab, followed by HDM-ASCT and lenalidomide maintenance [63]. Additionally, ongoing research suggests the early application of ASCT following induction therapy enhances outcomes [64–66]. With the introduction of new immunotherapies, including monoclonal antibodies and CAR-T cell therapy targeting MM cells, the role of ASCT may evolve, integrating these advances to improve response rates and minimize relapse [67, 68]. These newer strategies aim to reactivate the immune system, either passively or actively, providing deep and durable responses and raising the potential for their inclusion earlier in the treatment regimen [69]. For transplant-eligible newly diagnosed multiple myeloma (NDMM) patients, HDM plus ASCT remains the standard of care recommended by international guidelines from organizations such as the American Society of Clinical Oncology (ASCO), European Society for Medical Oncology (ESMO), and European Bone Marrow Transplantation (EBMT) [12, 63, 70]. Until recently, induction therapy typically involved a three-drug regimen of a proteasome inhibitor (PI), an immunomodulatory drug (IMiD), and dexamethasone. However, the treatment landscape has shifted with the introduction of anti-CD38 monoclonal antibodies, daratumumab and isatuximab, leading to the adoption of four-drug regimens (quadruplets) in place of the previous three-drug regimens (triplets) [71, 72].

#### Induction regimens for ASCT

Daratumumab, a human IgG/kappa monoclonal antibody targeting CD38, is approved for treating RRMM and NDMM [73–76]. It has become a new standard of care in transplant-eligible NDMM, as shown in the phase III CASSIOPEIA trial (NCT02541383), where adding daratumumab to VTd improved stringent complete response (sCR) rates and PFS, with 64% achieving minimal residual disease (MRD) negativity [77, 78]. The phase II GRIFFIN study (NCT02874742) further demonstrated the efficacy of daratumumab with VR, showing higher sCR and MRD negativity rates than VRd alone [79, 80]. These findings were confirmed by the phase III PERSEUS study (NCT03710603), where daratumumab plus VRd significantly improved PFS and MRD negativity rates [81]. Additionally, the phase II MASTER trial (NCT03224507) highlighted the potential of MRD-driven therapy adjustments with the Dara-KRd regimen (daratumumab, carfilzomib, lenalidomide, dexamethasone), offering a treatment-free state for MRD-negative patients [82]. These studies collectively affirm the benefits of incorporating daratumumab into standard treatment regimens for NDMM.

Isatuximab, a chimeric IgG monoclonal antibody targeting a unique epitope on CD38, exerts anti-myeloma effects through mechanisms including antibody-dependent cellular cytotoxicity (ADCC), complement-dependent cytotoxicity, antibody-dependent cellular phagocytosis, direct induction of apoptosis, and inhibition of CD38 enzyme activity [83, 84]. Approved for RRMM, it is also being explored for NDMM in transplant-eligible patients [85–88]. In the phase III GMMG-HD7 trial (NCT03617731), 660 transplant-eligible NDMM patients received either isatuximab plus bortezomib, lenalidomide, and dexamethasone (Isa-VRd) or standard VRd. The trial reported a 50% MRD negativity rate in the isatuximab group compared to 36% in the control group (p = 0.00017) [89]. The phase II GMMG-CONCEPT trial (NCT03104842) evaluated isatuximab with carfilzomib, lenalidomide, and dexamethasone (Isa-KRd) in high-risk NDMM patients. Post-consolidation, 72.8% achieved complete or stringent complete responses, 18.2% very good partial responses, and an overall response rate of 94.9%. MRD negativity was achieved by 67.7% after consolidation and 81.8% at some point. Sustained MRD negativity for 6 and 12 months was 72.7% and 62.6%, respectively. With a median follow-up of 44 months, the median PFS had not been reached, highlighting Isa-KRd's potential in high-risk NDMM [90].

#### Advancements in stem cell mobilization and collection techniques

Mobilizing CD34^+^ stem cells from bone marrow to peripheral blood is essential for harvesting adequate hematopoietic stem cells (HSC) for ASCT. A minimum of 2 × 10^6^/kg CD34^+^ cells is required, with optimal targets of > 3 × 10^6^/kg for one ASCT and > 6 × 10^6^/kg for two ASCTs. While the optimal mobilization strategy remains debated, current methods include granulocyte colony-stimulating factor (G-CSF), optionally preceded by Cyclophosphamide (1.5–4 g/m^2^) [91–93]. Plerixafor, a selective CXCR4 antagonist, enhances mobilization by preventing HSC adherence to the marrow, reducing procedure failure, and minimizing adverse events like neutropenia [94–96]. Anti-CD38 monoclonal antibodies used in induction therapy for NDMM have shown reduced HSC mobilization efficiency, as seen in the CASSIOPEIA trial, which reported lower CD34^+^ cell yields with the D-VTd regimen compared to VTd (6.7 vs. 10.0 × 10^6^/kg), higher plerixafor use (21.7% vs. 7.9%), and increased collection failures (24.6% vs. 11.4%) [97–99]. The MASTER and GRIFFIN trials also indicated high plerixafor use with daratumumab-containing regimens but found no negative impact on ASCT feasibility or safety [100]. Another study on 179 NDMM patients from the GMMG-HD6 and GMMG-HD7 trials (NCT02495922, NCT03617731) showed successful mobilization (> 6 × 10^6^/kg CD34^+^ cells) with VRd, I-VRd, and elotuzumab-VRd, confirming that isatuximab addition does not negatively affect mobilization [101–103]. These findings underscore that despite varying yields and increased plerixafor use, integrating daratumumab or isatuximab into induction regimens does not hinder successful stem cell collection or ASCT outcomes.

### T cell receptor (TCR) gene engineered T cells

The TCR is essential for the specific activation and clonal expansion of T cells in response to antigens. TCRs are generally heterodimers, composed of α and β chains, each featuring constant and variable domains. The variable domain undergoes somatic recombination, creating a vast diversity of TCR clonotypes, essential for recognizing antigens presented on cells by major histocompatibility complex (MHC) molecules [104, 105]. Unlike CARs, TCRs lack an intrinsic signaling domain and require the CD3 complex to transmit activation signals through phosphorylation of immunoreceptor tyrosine-based activation motifs (ITAMs). This interaction initiates various signaling pathways, leading to T cell activation [106, 107]. TCRs are highly sensitive; a few interactions with peptide-MHC (pMHC) complexes can trigger T cell responses, including cytokine production and target cell killing. High avidity TCRs, which bind strongly to pMHC, typically induce stronger immune responses and are more effective at lower antigen concentrations [108, 109]. However, T cells with lower-affinity TCRs are also crucial, maintaining immune effectiveness across a range of conditions and contributing to long-term immune memory, especially in chronic infections and cancer [110, 111]. Co-stimulation is necessary for optimal TCR signaling, provided by interactions with co-receptors and ligands that modulate T cell responses. This modulation influences T cell differentiation, proliferation, and longevity, which are key for effective immune responses and potential therapeutic interventions [109, 112, 113]. Understanding the integration of these signals can enhance T cell-based therapies by adjusting co-stimulation to improve T cell functionality in diverse disease settings.

TCR therapy for MM involves engineering patient T cells to target specific antigens such as NY-ESO-1 [37, 114], MAGE-A3 [115, 116], and BCMA [117], which are either unique to or overexpressed by MM cells. This strategy enables the modified T cells, upon reintroduction into the patient, to specifically identify and eradicate malignant cells. The effectiveness of this therapeutic approach largely depends on the selection of appropriate target antigens [67]. NY-ESO-1 is often selected due to its restricted expression in normal tissues, enhancing its safety profile [118, 119]. MAGE-A3 is chosen for its tumor-specific expression, providing a high degree of cancer selectivity [120, 121]. Additionally, BCMA, a target commonly utilized in CAR-T cell therapies [27, 122, 123], is increasingly being considered for TCR-based strategies due to its prevalent expression in MM cells [124]. However, BCMA itself, as a protein expressed on the surface of B cells, is not typically a target for TCR therapies because TCRs recognize peptides presented by MHC molecules on the surface of cells, rather than whole proteins or antigens directly exposed on the cell surface. This precision in antigen targeting is crucial for the success of TCR therapies in treating MM.

#### TCR‐engineered T cells targeting NY-ESO-1 tumor antigen

NY-ESO-1, a cancer-testis antigen, is primarily expressed in a variety of cancers but is absent in normal tissues with the exception of the testis. The testis lacks expression of the MHC, enabling evasion of immune detection. The restricted expression and immunogenic properties of NY-ESO-1 render it an ideal target for cancer immunotherapy [125–127]. In the context of oncological research, NY-ESO-1 mRNA has been identified in 20–40% of tumors, including those of the esophageal, gastric, melanoma, prostate, and several other carcinomas [128–131]. Its expression is notably associated with advanced cancer stages and correlates with poorer survival outcomes, emphasizing its potential as a prognostic marker. This antigen's relevance is particularly pronounced in MM, where NY-ESO-1 expression is predominantly observed in advanced stages of the disease [132]. The progression from diagnosis to relapse often sees an increase in NY-ESO-1 levels, mirroring broader malignancy trends where progression correlates with the upregulation of cancer/testis antigen (CTA) genes such as NY-ESO-1. This upregulation is likely a consequence of global hypomethylation events within the genome [133]. Mastaglio et al. demonstrated that single TCR gene editing using the clinical-grade HLA-A2 restricted NY-ESO-1157-165-specific TCR can quickly generate large quantities of tumor-specific T cells. These cells effectively target and eliminate cancer cells, showing a strong and safe performance compared to traditional TCR-transferred cells. The edited cells also have a better safety profile, minimizing risks like graft-versus-host disease in mice models. This approach offers a promising and safer method for advancing cancer immunotherapy treatments [118]. Moreover, Rapoport et al. conducted a study (NCT01352286) on 20 MM patients using engineered T cells targeted at cancer antigens NY-ESO-1 and LAGE-1. The treatment was well-tolerated, with no severe side effects, and the T cells showed effective targeting and persistence in the marrow. The presence of these T cells correlated with better clinical outcomes, leading to promising responses in 80% of the patients, with a median PFS of 19.1 months [37]. These studies confirmed that these engineered T cells are safe and effective for treating MM.

#### TCR‐engineered T cells targeting MAGE-A3 tumor antigen

MAGE-A3 is a member of the melanoma antigen gene (MAGE) family, which are typically not expressed in normal tissues except in testicular germ cells but are expressed in various types of cancers, including melanoma, non-small cell lung cancer, and others. This makes MAGE-A3 an attractive target for cancer immunotherapy [134–136]. Jungbluth et al. showed that MAGE-A3 serves as a promising antigen associated with myeloma, potentially valuable for vaccine-based immunotherapy. Additionally, the widespread expression and its association with cellular proliferation imply a pathogenic role for this gene in MM development [121]. Atanackovic et al. demonstrated that MAGE-A3 significantly enhances the survival of myeloma cells and their clonogenic precursors by diminishing the rates of both spontaneous and chemotherapy-induced apoptosis. Consequently, MAGE-A3 may serve as a promising target for the development of novel, myeloma-specific therapeutic strategies [120]. However, Linette et al. reported two patients with MAGE-A3-positive tumors received T cells modified to target an HLA-A*01-restricted peptide but died within a week from severe myocardial damage caused by an off-target reaction. Autopsies revealed no MAGE-A3 in the heart, but studies showed the T cells reacted to an unrelated cardiac peptide (NCT01350401 and NCT01352286) [137]. This highlights the risks of off-target effects with enhanced TCRs, emphasizing the need for careful analysis and early intervention in TCR-based therapies.

### Chimeric antigen receptor (CAR) T cells

**Fig. 3:**
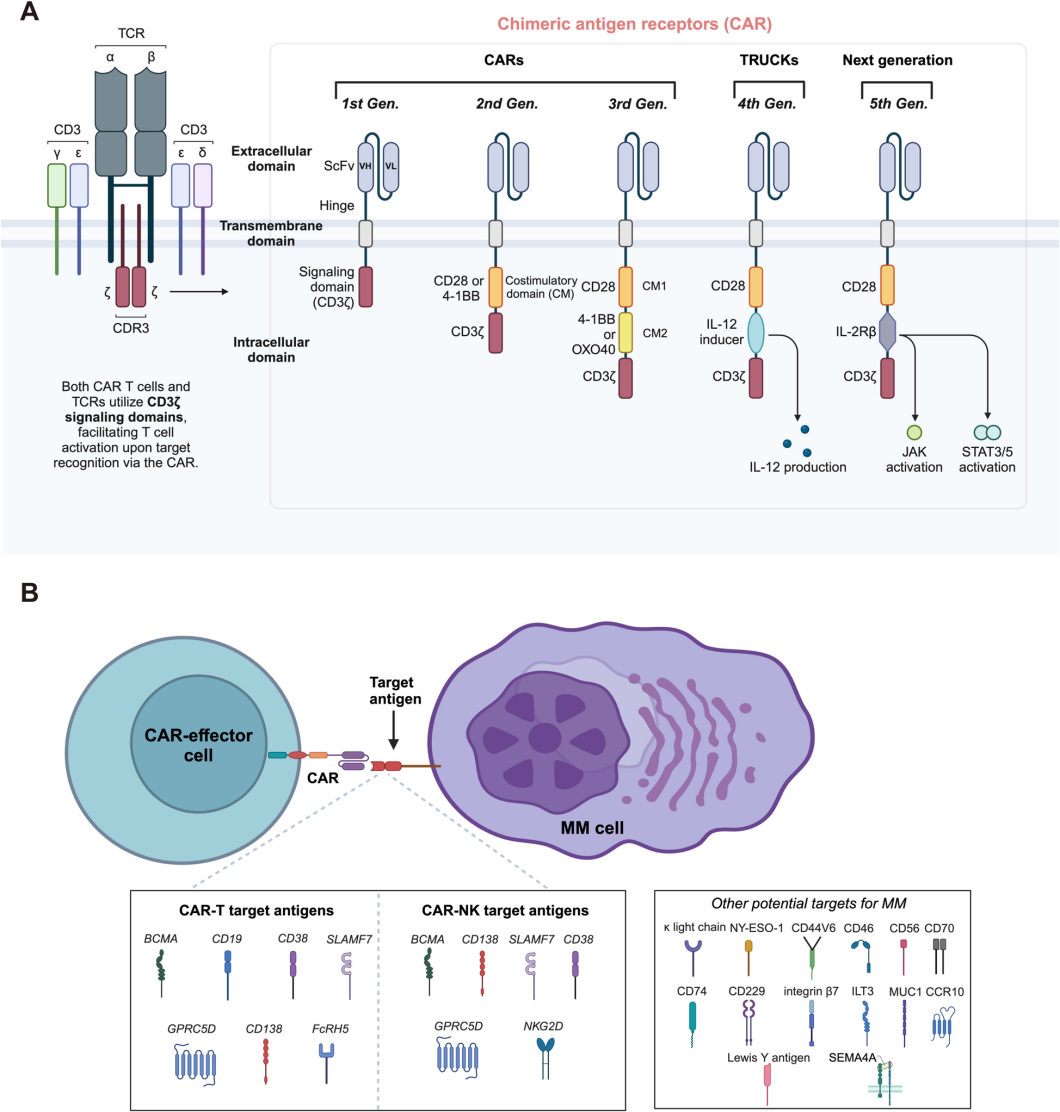
**A** Structure of different CAR generations. The core structure of a CAR is delineated by its primary components: the extracellular domain, the transmembrane domain, and the intracellular domain. The development of CAR-T cells has evolved significantly across generations. First-generation CARs featured only a signaling domain in the intracellular region. This was followed by second-generation CARs, which incorporated one co-stimulatory molecule. Third-generation CARs included an additional co-stimulatory molecule, enhancing their efficacy. Fourth-generation CARs, also known as TRUCKs (T cells redirected for universal cytokine-mediated killing), are based on second-generation designs but also express cytokines like IL-12 either constitutively or inducibly. The latest, fifth generation, builds further on the second-generation framework by adding intracellular domains of cytokine receptors, such as the IL-2Rβ chain fragment, which includes a STAT3/5 binding motif, to enhance signaling and T cell activity. **B** CARs can be engineered to be expressed on various immune cells, including T cells, NK cells, and macrophages, enabling these cells to recognize specific tumor antigens without reliance on MHC presentation. This overview encapsulates the array of molecules currently under investigation as potential CAR targets in MM. These include BCMA, CD19, CD38, CD138, SLAMF7, GPRC5D, FcRH5, NKG2D, k light chain, NY-ESO-1, CD44V6, CD46, CD56, CD70, CD74, CD229, integrin β7, ILT3, MUC1, CCR10, Lewis Y antigen and SEMA4A. Figure created with BioRender.com

CARs are genetically engineered receptors designed to recognize specific antigens, and are expressed on the surface of immune cells (Fig. 3B). The extracellular domain of a CAR typically consists of a single-chain variable fragment (scFv) that binds to antigens overexpressed on tumor cells. This scFv is connected via a hinge domain (e.g., CD8, CD28, IgG1, or IgG4) to a transmembrane domain (e.g., CD28, 4-1BB, or CD8). Intracellularly, CARs include one or more costimulatory domains (e.g., CD28, 4-1BB, or OX40, absent in first-generation CARs) and a CD3ζ activation domain [138, 139] (Fig. 3A). This configuration enables full T-cell activation upon antigen recognition. CAR-T cell therapy has significantly advanced cancer immunotherapy, demonstrating substantial efficacy in treating hematological malignancies, including MM [28, 140, 141].

#### BCMA targeted CAR‐T cells

BCMA, or B-cell maturation antigen is crucial in MM pathogenesis, largely due to its interactions with the ligands APRIL (a proliferation-inducing ligand) and BAFF (B-cell activating factor) [142–144]. These interactions support the survival and proliferation of MM cells [145]. BCMA is mainly found on plasma cells, which are central to MM, and is significantly overexpressed in myeloma cells compared to normal ones [122, 146]. Upon binding with APRIL and BAFF, BCMA triggers various signaling pathways, notably the NF-κB pathway, enhancing gene transcription that supports cell survival, growth, and chemotherapy resistance [147, 148]. This relationship also modifies the bone marrow environment, further facilitating myeloma cell growth. Given its specific overexpression in myeloma cells and limited presence in normal cells, BCMA is an effective target for therapies like antibody–drug conjugates, bispecific T-cell engagers (TCEs), and CAR-T cell therapies [27, 67, 149, 150]. These treatments focus on selectively eliminating myeloma cells with minimal impact on healthy cells.

In the preclinical phase, BCMA targeted CAR-T cells demonstrated potent cytotoxic activity in vitro and in vivo against myeloma cells. These studies often involve testing the CAR-T cells against human myeloma cell lines in mice models to observe the efficacy and safety of the treatment [151–156]. Safety is a critical aspect of preclinical trials. CAR-T cell therapy can lead to CRS and neurotoxicity, which are significant concerns [157, 158]. Preclinical models have been used to study these effects and refine the cell manufacturing process and dosing strategies to minimize adverse effects. Moreover, further advancements in CAR-T cell designs are ongoing in preclinical studies to enhance their effectiveness and reduce side effects. This includes modifications like the inclusion of suicide genes [159], dual-targeting CARs [160], or using different co-stimulatory domains to improve cell persistence and function [161, 162]. These preclinical findings are foundational for advancing BCMA targeted CAR-T therapy into clinical settings, where the real-world efficacy and safety can be evaluated in patients with MM. This research is crucial in potentially offering a new and effective treatment for patients who have RRMM.

In the clinical phase, Idecabtagene vicleucel (Abecma), also known as Bb2121, is the first CAR-T cell therapy approved by the U.S. Food and Drug Administration (FDA) for adults with RRMM in March 2021. This therapy targets patients whose MM has either recurred or failed to respond to prior treatments [163]. Lin et al. conducted a phase I multicenter study (CRB-401) on 62 patients with RRMM, followed for a median of 18.1 months. The study (NCT02658929) primarily assessed safety and showed low rates of serious side effects, with 6.5% experiencing severe CRS and 1.6% severe neurotoxicity. The overall response rate (ORR) was 75.8%, with 64.5% achieving a very good partial response (VGPR) or better, and 38.7% reaching CR or sCR. Secondary measures included median durations of CR, PFS, and OS at 10.3, 8.8, and 34.2 months, respectively. Furthermore, the expansion of Bb2121 in blood and bone marrow was linked to clinical effectiveness and a decrease in soluble BCMA. Notably, patients with longer PFS (≥ 18 months) had T cells that were less exhausted and showed a more robust functional phenotype [38]. These findings support the safety, tolerability, and effectiveness of Bb2121, highlighting specific T cell characteristics associated with durable responses. LCAR-B38M (JNJ-68284528) is a second-generation, bispecific CAR-T cell therapy targeting two distinct BCMA epitopes, enhancing its binding affinity. This differentiates it from other BCMA-directed CAR-T therapies. The therapy is being evaluated in the LEGEND-2 phase I/II trial (NCT03090659) in China for patients with RRMM. The study reported a 5-year PFS of 21.0% and OS of 49.1%, noting a stabilization of survival curves over time. Participants achieving CR demonstrated notably higher 5-year PFS and OS rates of 28.4% and 65.7%, respectively. Notably, 12 patients (16.2%) maintained relapse-free status despite high-risk cytogenetic abnormalities and all had restored normal humoral immunity. A sustained CR was associated with favorable prognostic factors including good performance status, IgG subtype, absence of extramedullary disease, and a lymphodepletion regimen combining cyclophosphamide (CTX) and fludarabine (FAM). Among the patients, 83.8% experienced disease progression or death; however, 61.1% of those responded to subsequent therapies, particularly proteasome inhibitor-based treatments. Safety profiles showed comparable recovery of hematologic and hepatic functions across groups, with a low incidence of secondary malignancies (5.4%) and no severe viral infections. One patient in persistent remission exhibited a sustainable CAR-T population characterized by a predominance of indolent, low-cytotoxicity CD4/CD8 double-negative T cells [39]. The CARTITUDE-1 phase Ib/II study (NCT03548207) evaluated ciltacabtagene autoleucel (cilta-cel) in patients with relapsed/refractory multiple myeloma, demonstrating sustained, profound responses after 12 months. Updated results, with a median follow-up of 27.7 months (N = 97), show a remarkable ORR of 97.9% and a sCR rate of 82.5%. Patients, including high-risk subgroups, received a single cilta-cel infusion following lymphodepletion. Median PFS and OS were not reached, with 27-month PFS and OS rates at 54.9% and 70.4%, respectively. Response durations were reduced in high-risk patients. The treatment's safety profile remained manageable, with stable adverse events. These findings, at around 28 months, confirm cilta-cel's robust efficacy and favorable risk/benefit ratio in treating advanced MM [40]. In a phase Ib study (NCT02546167), Cohen et al. evaluated autologous T cells modified with a lentiviral BCMA-specific CAR, incorporating CART-BCMA, in 25 patients with RRMM. Participants were assigned to three cohorts: 1) 1–5 × 10^8^ CART-BCMA cells, 2) 1–5 × 10^7^ CART-BCMA cells plus CTX at 1.5 g/m^2^, and 3) 1–5 × 10^8^ CART-BCMA cells plus CTX at the same dose. BCMA expression was not a criterion for inclusion. All patients received the engineered T cells, which expanded successfully. Significant adverse effects were CRS and neurotoxicity, observed as grade 3–4 in 32% and 12% of patients, respectively, but these were reversible. One death occurred due to candidemia and advancing MM on day 24 after severe complications. Therapeutic responses varied across cohorts, with efficacy rates of 44% in cohort 1, 20% in cohort 2, and 64% in cohort 3. Outcomes included five partial, five very good partial, and two complete responses. Three responses were maintained for up to 32 months. Response and T cell expansion correlated with the CD4:CD8 ratio and CD45RO^−^CD27^+^CD8^+^ T cell prevalence in the pre-treatment leukapheresis product. CART-BCMA treatment, with or without lymphodepleting chemotherapy, showed clinical activity in extensively treated MM patients [41]. The phase I dose-escalation trial (NCT03070327) assessed MCARH171 in RRMM patients. Participants underwent a FAMP and CTX conditioning regimen before receiving 1–2 doses of MCARH171. Four escalating doses tested range from 72 × 10^6^ to 818 × 10^6^ CAR-T cells. As of July 16, 2018, 11 patients, having previously failed an average of six myeloma treatments, were treated, achieving an ORR of 64% with a median duration of response (mDOR) of 106 days. Patients in high-dose cohorts exhibited greater peak expansion, prolonged persistence of MCARH171, and more sustained clinical responses compared to those in low-dose cohorts. CRS was reported in six patients, with two experiencing grade 3 severity. Additionally, one patient developed transient grade 2 encephalopathy that resolved within 24 h. No dose-limiting toxicities (DLTs) were observed [42]. CART-BCMA therapies, have demonstrated significant efficacy in RRMM, though they are associated with severe neurological toxicities, primarily immune effector cell-associated neurotoxicity syndrome (ICANS). ICANS typically occurs within the first week of treatment and presents with a range of symptoms, including headache, confusion, delirium, aphasia, tremors, and seizures. In more severe cases, it can progress to encephalopathy, coma, or cerebral edema, which may be fatal. Motor dysfunction, such as tremors and muscle weakness, and seizures have also been reported. While ICANS often occurs alongside CRS, it can manifest independently. Prompt recognition and management of these neurotoxic effects, typically with corticosteroids or other immunosuppressive agents, are critical to preventing life-threatening complications [164]. Despite these risks, studies consistently highlight the potential of BCMA-targeted CAR-T therapies in advanced MM, demonstrating both robust efficacy and a manageable safety profile across diverse patient cohorts.

#### CD19 targeted CAR‐T cells

CD19 is typically not targeted in MM treatments because it is mainly found on B cells and their precursors, while MM primarily arises from plasma cells, which do not usually express CD19 [165–168]. Consequently, CD19-targeted CAR-T cell therapy, effective in other B-cell malignancies, has limited applicability in MM [169, 170]. However, emerging research indicates that a small subset of MM cells might express CD19 or that combining CD19 with other antigens could enhance treatment efficacy [28]. The infusion of anti-CD19 and anti-BCMA CAR-T cells in patients with NDMM or RRMM has shown promising results and manageable side effects [43, 44], despite not conclusively preventing progression post-anti-BCMA therapy. There are indications of its potential benefit, particularly in dual-targeted strategies. For instance, early studies have begun to explore dual-targeting CAR-T cells aimed at both BCMA and CD19, though these are less prevalent than BCMA-focused therapies [171]. A recent Phase I/II trial in China (ChiCTR2000033567) investigating a BCMA-CD19 bispecific CAR-T cell therapy showed that BC19 CAR T cells are feasible, safe, and effective for treating patients with RRMM, demonstrating promising early responses [45].

#### CD38 targeted CAR‐T cells

CD38, a glycoprotein found abundantly on the surface of MM cells, serves as an ideal target for therapeutic interventions, such as monoclonal antibodies (daratumumab and isatuximab) [83, 172–174]. This molecule is integral to various cellular processes including cell adhesion, signal transduction, calcium signaling, and the regulation of apoptosis—a key mechanism in cancer treatment [175, 176]. Its high and uniform expression on plasma cells and other lymphoid cells highlights its potential as a focal point for novel therapeutic strategies in MM [177, 178]. Several research groups have developed anti-CD38 CAR-T cells and tested them in preclinical studies [179–182]. These cells often lack CD38 expression, likely due to the elimination of CD38-positive cells among them, a process known as fratricide. Despite this, the anti-CD38 CAR-T cells effectively target myeloma cells, supporting previous findings that CD38 is not essential for T cell functionality [183]. Notably, Glisovic-Aplenc et al. reported the anti-CD38 CAR-T cells they produced did not experience fratricide, potentially because of a protective mechanism within the CAR construct. These CAR-T cells can deplete CD34^+^ CD38^+^ hematopoietic progenitors in vitro and in vivo; however, they appear to spare other hematopoietic lineages, indicating that the CD34^+^CD38^−^ low/negative cells can sustain hematopoiesis [180, 182]. As these therapies progress to clinical trials, it is crucial to monitor their impact on the immune and hematopoietic systems in patients.

In the clinical phase, Mei et al. developed a CAR-T cell with dual targeting domains for CD38 and BCMA, and a 4-1BB co-stimulatory domain, selecting a scFv with lower CD38 affinity to minimize hematopoietic toxicity (ChiCTR1800018143) [46]. This construct was administered to 23 patients, resulting in an ORR of 87%, with 52% achieving CR. Common toxicities included CRS in 87% of patients and significant cytopenias in 96%, with severe cases (grade ≥ 3) in 17% and 87% respectively. Two fatalities occurred due to infection and cerebral hemorrhage. The duration of response (DOR) reached 76% over one year. Another study with a similar dual-targeted CAR-T cell construct reported on 16 RRMM patients, showing comparable toxicities and an ORR of 88% with 81% CR. Notably, one patient died from an infection during prolonged CRS and persistent cytopenias related to hemophagocytic lymphohistiocytosis [47]. Moreover, Zhang et al. administered separate anti-CD38 and anti-BCMA CAR-T cells to 22 patients, achieving an ORR of 91% and CR rate of 55%. However, two deaths occurred due to CRS [48]. In summary, the primary challenge in analyzing these studies is determining the specific toxicity and responses caused by the anti-CD38 therapy component, as it was always used in combination with anti-BCMA constructs.

#### SLAMF7 targeted CAR‐T cells

The glycoprotein cell surface receptor signaling lymphocytic activation molecule family member 7 (SLAMF7), also known as CD319 or CS1, is a receptor found primarily on MM cells, natural killer cells, and some T cell subsets [184, 185]. Its high expression on malignant plasma cells and crucial role in plasma cell survival has led to the development of targeted therapies [186]. One such therapy, the anti-SLAMF7 antibody elotuzumab, has been FDA-approved for use with lenalidomide and dexamethasone in treating RRMM [187]. Additionally, using CAR-T cells to target SLAMF7 offers a promising approach to treat MM.

Several groups have developed anti-SLAMF7 CAR-T cells, showing promise in preclinical models for treating MM. Gogishvili et al. engineered CAR T cells with elotuzumab's target-binding domain and a CD28 co-stimulatory domain, effectively targeting myeloma in patient-derived and murine models [188]. Although SLAMF7 is also present on various immune cells, leading to potential fratricide, post-manufacture CAR T cells mostly lacked SLAMF7 expression, mitigating this issue. They also spared SLAMF7-low immune cells while depleting high expressers. Furthermore, Roders et al. enhanced anti-myeloma efficacy by using CRISPR/Cas9 to eliminate CD38 in T cells, creating a dual CAR system targeting CD38 and SLAMF7 [189]. This approach showed robust responses without the toxicity seen in anti-CD38 CAR T therapy, suggesting a safer alternative. O'Neal et al. utilized a different SLAMF7-binding epitope and a third-generation co-stimulatory domain, producing mainly CD4^+^ CAR-T cells due to CD8^+^ T cell fratricide [190]. They further applied CRISPR/Cas9 to prevent fratricide, achieving a balanced CD4/CD8 ratio without enhancing efficacy significantly. Collectively, these studies indicate the potential of anti-SLAMF7 CAR-T cells in myeloma treatment, though the implications of fratricide require more research. In a phase I/IIa clinical trial initiated based on preclinical studies of a dual-targeted single-chain CAR featuring anti-BCMA and anti-SLAMF7 domains, results from 16 treated patients were recently published [49]. The trial reported toxicities including CRS in 38% of cases, 6% of which were grade 3 or higher, but no instances of Immune effector cell-associated neurotoxicity syndrome (ICANS). All patients experienced cytopenias, with 100% encountering severe (grade ≥ 3) cases. Infections occurred in 38% of the patients, with severe infections (grade ≥ 3) in 31%. Efficacy was notable, with an ORR of 81% and sCR rate of 38%.

#### GPRC5D targeted CAR‐T cells

G protein-coupled receptor class C group 5 member D (GPRC5D) is a protein predominantly expressed on the surface of MM cells but with limited expression in normal tissues [191–193]. This makes it an attractive target for CAR-T therapy because therapies directed against it can potentially kill myeloma cells while sparing most healthy cells [194]. In murine and nonhuman primate models, CAR-T cells targeting GPRC5D showed effective anti-MM activity in vivo, including in BCMA escape models, without on-target, off-tumor toxicity [195, 196]. This success has spurred the clinical development of therapeutic agents that target GPRC5D for MM treatment. In a 2022 phase I study by Mailankody et al. [50], 17 RRMM patients, all previously treated with at least three lines of therapy including proteasome inhibitors (PIs), IMiDs, anti-CD38, and BCMA-targeted therapies, received infusions of MCARH109. This CAR-T cell therapy features a humanized anti-GPRC5D scFv target-binding domain and a 4-1BB co-stimulatory domain. Doses ranged from 25 to 450 × 10^6^ CAR T-cells. The study established 150 × 10^6^ CAR-T cells as the maximum tolerated dose after observing severe adverse events, including grade 4 CRS and ICANS in one patient, and grade 3 cerebellar disorder in two patients at the highest dose level. These neurological effects were likely due to low-level, off-target GPRC5D expression. Among patients who received 25 to 150 × 10^6^ CAR-T cells, no severe CRS or neurotoxicity was reported, and ORR was 71%, with 58% for those administered up to the maximum tolerated dose. Other mild side effects included grade 1 nail changes in 65% of patients and grade 1 taste alterations or dry mouth in 12%. Moreover, BMS-986393 is an autologous CAR-T cell therapy targeting GPRC5D, evaluated in a phase I, first-in-human trial (CC-95266-MM-001, NCT04674813) [51]. This multi-center study involved patients with three or more prior lines of therapy, including PIs, IMiDs, anti-CD38 therapy, and ASCT, alongside previous BCMA-targeted therapies. In the dose expansion cohort of 70 patients, BMS-986393 doses ranged from 25 to 450 × 10^6^ CAR-T cells. Of these patients, 46% had prior BCMA-targeted therapy, and 36% had prior BCMA-directed CAR-T cell therapy. The ORR was 86%, with a 38% CR rate in patients with assessable efficacy, and 85% ORR with a 46% CR in those refractory to prior BCMA-targeted therapies. Common severe side effects included neutropenia (69%), anemia (31%), and thrombocytopenia (30%). There were no severe adverse events related to skin, nails, or taste. CRS occurred in 84% of patients, mostly mild; however, severe CRS led to one death and affected three additional patients. Neurological toxicities were noted, with 11% experiencing ICANS, and other neurological symptoms like cerebellar toxicity and headache occurring in a few patients. This data supports the potential of BMS-986393 as a treatment for RRMM, with further investigations ongoing. Furthermore, OriCAR-017, another GPRC5D-targeted autologous CAR T-cell therapy, features the proprietary Ori signal activation domain to enhance memory immune cells' expansion efficiency, boosting the anti-tumor effectiveness and longevity of CAR T-cells in vivo [191]. In the phase I POLARIS trial in China (NCT05016778) [52], 10 RRMM patients received OriCAR-017 in doses from 1 to 6 × 10^6^ CAR-T cells/kg. All patients experienced hematologic toxicities, such as neutropenia (100%), thrombocytopenia (90%), leukopenia (90%), and anemia (70%). Ninety percent encountered grade 1 CRS, 10% had grade 2 CRS, and there were no cases of neurologic toxicities. ORR was 100%, with 60% achieving sCR. After a median follow-up of 7.8 months, disease progression occurred in two patients. However, mechanisms underlying resistance to anti-GPRC5D CAR-T cell therapy are becoming clearer. Mailankody et al. showed that unlike relapse after anti-BCMA CAR-T cell therapy, where BCMA loss is rare, four out of six patients who initially responded and then relapsed showed complete loss of GPRC5D expression. Notably, one patient exhibited biallelic deletions at the GPRC5D loci [50]. Additional studies have identified complex GPRC5D deletions and mutations during relapse following anti-GPRC5D bispecific TCE therapy, pointing to genetic alterations that reduce GPRC5D expression [197]. Moreover, Derrien et al. reported a patient with decreased chromatin accessibility at the GPRC5D promoter and distant enhancer regions, suggesting epigenetic silencing [198]. These findings underscore the intricate tumor biology of myeloma and the need for comprehensive treatment strategies to overcome resistance.

GPRC5D-targeted therapies have shown promise, particularly for patients who have previously failed BCMA therapies, offering reduced infection risks [194]. Common side effects include skin and oral issues such as rash and dry mouth, which are generally manageable with standard care, though taste changes remain a challenge. These therapies are associated with fewer such side effects compared to TCE therapies, possibly due to different tissue distributions and dosing regimens [199]. Some unique side effects of CAR-T cell therapies include dizziness at high doses. Recent studies, including dual-targeted BCMA and GPRC5D therapies, suggest potential for significant improvements in treatment outcomes for MM, highlighting GPRC5D's role in advancing MM treatment strategies [200]. Future clinical trials and novel approaches like dual-targeting constructs and combination therapies are currently being explored, indicating a robust pipeline for enhancing therapeutic efficacy and safety.

#### CD138 targeted CAR-T cells

CD138 (Syndecan-1), a transmembrane proteoglycan, is primarily expressed on terminally differentiated B cells and is essential for plasma cell survival [201, 202]. However, its expression on other cell types such as epithelial and endothelial cells theoretically limits its utility as a therapeutic target [203]. Despite these challenges, anti-CD138 CAR-T cells have been developed and preclinically tested. These cells, as demonstrated by Sun et al., did not affect endothelial or epithelial cells in co-culture experiments [204]. Ongoing clinical and preclinical studies, including a U.S. trial (NCT03672318), aim to optimize this therapy. Notably, a novel dual-split CAR construct targeting both CD38 and CD138 antigens showed efficacy in eliminating malignant plasma cells while sparing hematopoietic precursors. Additionally, a phase I trial (NCT01886976) involving a CD138-directed CAR-T cell with a 4-1BB domain in RRMM patients reported manageable side effects and detectable CAR-T cells up to three months post-treatment. Despite CD138 expression in normal tissues, no off-target effects have been reported in ongoing trials, though the limited efficacy of these constructs raises questions about the potential safety of more potent CD138-targeted therapies.

#### FcRH5 targeted CAR-T cells

Fc receptor-homolog 5 (FcRH5) is predominantly expressed on plasma cells, marking it as a promising target for MM immunotherapy [205]. Its expression is limited primarily to certain B cell subsets and is notably heightened in MM patients with a 1q21 amplification, a known adverse prognostic factor [206]. Cevostamab, a bispecific TCE targeting FcRH5, is currently in early clinical trials and has demonstrated promising efficacy with minimal toxicity [207]. Additionally, preclinical developments include an anti-FcRH5 CAR-T cell therapy that effectively eradicates myeloma cells both in vitro and in vivo [208]. This includes a model of myeloma resistant to BCMA-targeted therapies. A dual-targeted CAR-T therapy combining anti-BCMA and anti-FcRH5 has also shown potential. While no clinical trials for anti-FcRH5 CAR-T cells are ongoing, their future exploration is anticipated. However, more comprehensive clinical data are required to fully assess the safety and efficacy of FcRH5-directed therapies.

#### Other potential targets for MM

Several clinical trials have evaluated other targeted CAR-T cell therapies in myeloma with limited success. Trials with anti-κ light chain CAR-T cells aimed at the κ light chain found in many B cell tumors showed no positive responses in myeloma patients [209]. Similarly, trials using anti-NKG2D ligand CAR-T cells, which target widely expressed NKG2D ligands on various tumors, also failed to show effectiveness [210, 211]. Additionally, trials with anti-NY-ESO-1 TCR-engineered T cells post-ASCT indicated some biological activity, but the results were mixed and the effectiveness of the CAR-T cells themselves remains unclear [37, 212]. Preclinical studies have also identified several other potential targets for CAR-T cell therapy in myeloma, including CD44 splice variants [213], CD46 [214], CD56 [215], CD70 [216], CD74 [217], CD229 [218, 219], integrin β7 [220], Lewis Y antigen [221], ILT3 [222], SEMA4A [223], CCR10 [224], and Mucin 1 (MUC1) [225].

### CAR-NK cells or genetically engineered NK cells

Engineering of natural killer (NK) cells has emerged as a promising cancer therapy, offering an alternative to conventional methods [226–228]. NK cells, which are part of the innate immune system, can be activated without antigen presentation or strict matching of human leukocyte antigens (HLAs), unlike T cells. This allows the development of CAR-NK cells, which are less likely to induce graft-versus-host disease (GVHD), making them suitable for "off-the-shelf" use [229, 230]. CAR-NK cells can be sourced from established NK cell lines like NK92 or from induced pluripotent stem cells (iPSCs), bypassing the need for cells from the actual patient [231, 232]. Additionally, NK cells kill cancer cells by releasing perforin and granzyme, and expressing ligands such as FasL and TRAIL, significantly reducing the risk of CRS often associated with CAR-T cell therapies [233, 234]. NK cells can be derived from various sources, including peripheral and cord blood, as well as iPSCs, allowing for allogeneic use that does not require donor-patient HLA matching. This versatility could potentially lower the costs of CAR cell therapies. CAR-NK therapy is appealing because it is less likely to cause CRS and GVHD and can counteract the tumor's resistance mechanisms [235]. However, challenges remain, such as lower transduction efficiency and expansion issues, particularly with peripheral blood-derived NK cells. Cord blood-derived NK cells tend to minimize these problems but are relatively immature, which is a drawback [236, 237]. NK92, an IL-2-dependent immortalized cell line derived from a lymphoma patient, requires irradiation before clinical use due to safety concerns, despite the general safety of infusion. The primary advantages of NK92 are its ease of expansion and availability, which reduce both treatment initiation time and costs. However, while NK-92 cell lines are readily manipulable and expandable, they pose safety risks and exhibit poor long-term survival [238, 239]. Enhancing the survival, cytotoxicity, and tumor-targeting of CAR-NK cells are critical areas of ongoing research in improving the effectiveness of CAR-NK cell therapies.

Ren et al. and Cao et al. developed BCMA-specific CAR-NK cells targeting MM, enhancing cytotoxicity and survival in mouse models [240, 241], with ongoing clinical trials (NCT03940833 and NCT05182073) exploring their therapeutic potential. Jiang et al. demonstrated that CD138-specific CAR-NK cells target CD138-positive malignancies, potentially improving remission outcomes post-chemotherapy [242]. Chu et al. advanced SLAMF7-specific CAR-NK cell therapy, showing significant tumor inhibition and survival extension in MM models, indicating its promising treatment prospects [243]. Additionally, studies revealed that NKG2D-CAR NK cells, engineered from autologous NK cells of MM patients, safely enhance antimyeloma activity [244]. Reiser et al. developed the iPSC-derived FT555 CAR-NK cell product targeting GPRC5D and CD38, used alongside daratumumab, providing a scalable, off-the-shelf therapeutic option for broad MM patient access [245]. These innovations highlight significant advances in NK cell therapies for MM, focusing on dual targeting and engineered enhancements to improve efficacy and patient outcomes.

CAR-NK cell therapy, inspired by CAR-T methods, requires sophisticated cell processing facilities and trained personnel. Optimizing CAR properties and NK cell metabolism is key to combating drug-resistant MM. NK cells, with their inherent anti-tumor abilities, are enhanced to improve lifespan and activation for better MM response. CAR-NK targets multiple stable antigens to avoid issues like antigen shedding and off-target effects seen with CAR-T therapies. Additionally, off-the-shelf NK cell therapies are being developed to reduce costs and widen patient access. Unlike T cell therapies, repeated NK cell doses are necessary for a sustained and effective anti-MM response, offering a promising alternative for improving MM patient outcomes.

### Bi- and trispecific immune cell engagers for cell therapy of MM

Bi- and trispecific T cell and NK cell engagers are emerging targeted immunotherapies aimed at enhancing the antitumor response against MM [246–249]. These molecules typically consist of single-chain variable fragments that bind simultaneously to CD3 on T cells and a tumor-associated antigen like BCMA or CD19, commonly overexpressed in MM cells [149, 250]. By forming an immunological synapse between T cells and cancer cells, these engagers facilitate targeted tumor cell killing. Trispecific engagers further enhance this approach by incorporating an additional binding domain, boosting specificity and immune attack potency [251]. NK cell engagers activate NK cells by targeting receptors such as CD16, alongside a tumor-specific antigen, directing NK cell cytotoxicity towards MM cells [252–254]. These dual and triple targeting strategies amplify the immune response and mitigate antigen escape, a common challenge in MM treatment [140, 255, 256]. However, these engagers can induce severe side effects like CRS, necessitating ongoing optimization to balance efficacy with safety [246, 257]. Current clinical trials are promising, indicating potential in achieving sustained responses in MM, particularly in cases resistant to conventional treatments [258]. Integrating these novel engagers with other therapies could enhance outcomes through a robust, precisely targeted immune approach.

As mentioned previously, BCMA is a crucial target in MM treatment due to its role in cell proliferation and survival. It is primarily expressed on malignant and normal plasma cells, but not on hematopoietic stem cells or most non-hematopoietic tissues, making it an ideal target for T cell-redirecting therapies. Elevated levels of soluble BCMA (sBCMA) are associated with disease progression. The FDA has approved several BCMA-targeted therapies, including CAR-T products Abecma and cilta-cel, and the antibody–drug conjugate belantamab mafodotin, which was withdrawn in 2022 after failing a phase III trial [259]. In October 2022, subcutaneous teclistamab was approved for patients with RRMM who had previously failed multiple treatments, marking it the first anti-BCMA × anti-CD3 TCE bispecific antibody (BsAb) to receive approval [260]. Teclistamab showed an ORR of 63% and CR rate of 39.4% in clinical trials (NCT03145181, NCT04557098). Despite a lower response rate compared to some CAR-T treatments, teclistamab offers a safer profile and easier production [261, 262]. Other promising BCMA-targeted BsAbs like elranatamab and linvoseltamab are undergoing FDA review or clinical trials with favorable preliminary results [263, 264]. Emerging treatments for MM include talquetamab and cevostamab. Talquetamab targets GPRC5D, a novel receptor expressed on MM cells. Cevostamab (RG6160) is an FcRH5 × CD3 TCE that binds to a membrane-proximal epitope of FcRH5, promoting efficient synapse formation and MM cell killing. Clinical studies have demonstrated high efficacy for both treatments [265, 266]. Other strategies include targeting CD38 and SLAMF7 with TCE BsAbs and exploring trispecific antibodies (TsAbs) combining multiple targets for enhanced efficacy [246].

It is worth noting that redirecting NK cells to kill tumors is a potential alternative to T cell based therapies, which, though effective, often cause severe side effects like CRS. Clinical responses observed with anti-CD19 CAR-NK cells, without major toxic effects, illustrate the potential of NK cell based immunotherapy [267]. Most NK cell engagers (NKCEs) display an antibody fragment directed against CD16a, similar to the CD3-targeting moiety of TCE [268]. NKCEs like AFM13, a chimeric tandem diabody (TandAb) with anti-CD30 and anti-CD16a domains, have shown potent ADCC and promising results in clinical trials, especially when combined with allogenic NK cells [269]. Advanced NKCEs such as antibody-based NKCE technology and trispecific NKCE therapies platforms incorporate multiple binding domains to enhance NK cell activation and tumor cell killing. For example, the trispecific NKCE (IPH6401/SAR445514) targets BCMA, NKp46, and CD16a, showing potent antitumor activity in preclinical studies and ongoing phase I trials [270]. IL-15-based trifunctional NK cell engagers (TriKEs) like GTB-5550 enhance NK cell activation and proliferation, showing promising preclinical results in treating MM [271]. Overall, bi- and trispecific T cell and NK cell engagers represent a significant advancement in MM therapy, offering targeted, potent, and potentially safer alternatives to existing treatments. Ongoing clinical trials and optimization efforts are crucial to fully realizing their therapeutic potential and integrating them into standard MM treatment regimens.

### Other adoptive cell therapies for MM

#### Lymphokine-activated killer (LAK) cells

Lymphokine-activated killer (LAK) cells, primarily derived from NK cells and T-lymphocytes, are activated by interleukin-2 (IL-2) and exhibit potent cytotoxic activity against tumor cells [272, 273]. LAK cells express NK markers such as CD3^−^CD56^+^ and NKG2D, allowing for HLA-independent killing mechanisms [274, 275]. A phase I/II trial assessed low-dose recombinant interleukin-2 (rIL-2) in advanced MM patients who failed standard chemotherapy [53]. Eighteen patients received subcutaneous rIL-2. Tumor response occurred in 6 of 17 patients: 2 had tumor reduction, and 4 achieved stable disease. Eosinophil counts increased 15-fold, CD4^+^ T cells activated, and CD56^+^ NK cells expanded. The CD4^+^/CD8^+^ ratio normalized, and NK/LAK cell activities enhanced. Endogenous rIL-2 production and soluble rIL-2 receptor levels also increased. In another clinical trial, 16 patients received rIL-2 and LAK cells to reduce relapse rates after autologous bone marrow transplantation (ABMT) [54]. Common side effects included fever, nausea, and rash. Dose-limiting but reversible toxicities were hypotension and thrombocytopenia. Higher rIL-2 doses enhanced NK and LAK cell activity, indicating a strong immunomodulatory effect. These results suggest that rIL-2 and LAK cells warrant further investigation for reducing relapse in advanced hematological malignancies. While low-dose rIL-2 can boost immune function in MM, its efficacy is limited in advanced stages due to tumor-induced immunodeficiency. Future studies should explore the role of rIL-2 in maintaining remission post-chemotherapy. Interestingly, Gottlieb et al. found rIL-2 enhanced cytotoxicity in plasma cell lines and malignant cells from MM patients [276]. Healthy donors' PBMCs showed minimal killing ability, increasing slightly with rIL-2. MM patients' PBMCs induced significant lysis of malignant cells post-rIL-2 exposure. rIL-2-stimulated monocytes released TNF and interferon-γ (IFNγ), reducing malignant cell survival in culture. In vivo, four MM patients received seven rIL-2 courses post-ABMT without serious side effects. rIL-2 increased NK and LAK cell activities and TNF and IFNγ production. These results suggest rIL-2 administration in MM warrants further evaluation, especially for controlling minimal residual disease. However, LAK cell therapy has been replaced by more specific immunotherapies [277, 278].

#### γδ T cells from TILs and PBMCs

**Fig. 4:**
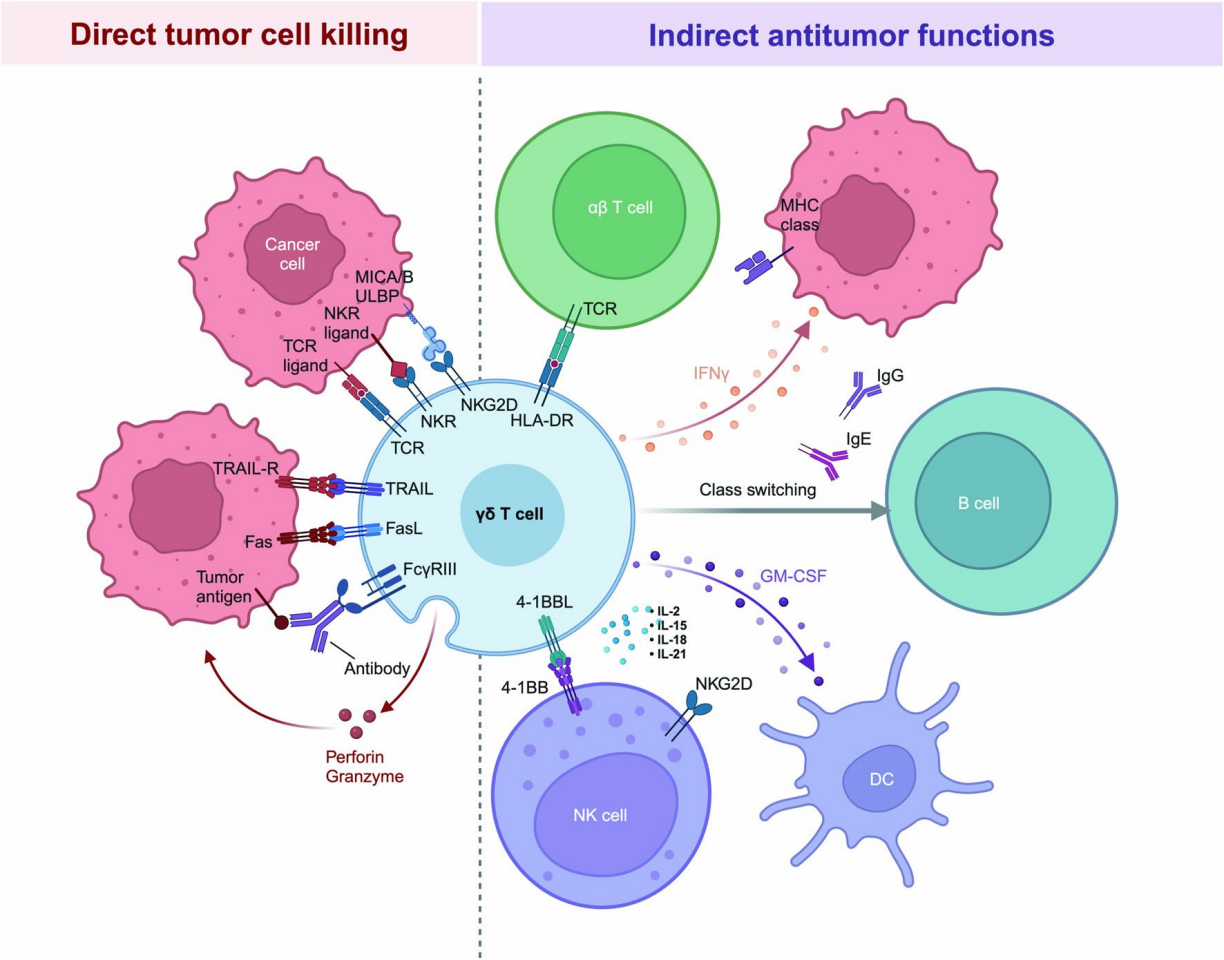
Antitumour γδ T cell functions and their regulation. γδ T cells recognize and kill tumor cells via their TCRs and NKRs, mediating tumor cell killing through TRAIL, FASL, and the granule exocytosis pathway, which involves perforin and granzyme secretion. Additionally, they engage in antibody-dependent cellular cytotoxicity when tumor-specific antibodies are present. γδ T cells enhance antitumor immune responses by producing IFNγ and acting as antigen-presenting cells, which activate αβ T cells. They also express the 4-1BBL to stimulate NK cells and induce antibody class switching in B cells, bolstering the humoral response. Moreover, γδ T cells produce GM-CSF to regulate DC infiltration. The antitumor activity of γδ T cells is further enhanced by IL-2, IL-15, IL-18, and IL-21. FcγRIII, Fcγ receptor III; HLA-DR, human leukocyte antigen-DR; LDL, low-density lipoprotein; MHC, major histocompatibility complex; NKG2D, natural killer group 2D; TRAIL-R, TRAIL receptor. Figure created with BioRender.com

Γδ T cells, a distinct subset of T cells abundant in mucosal organs, constitute less than 5% of peripheral blood lymphocytes [279–281]. They are non-HLA-restricted cytotoxic cells playing a crucial role in both innate and adaptive immunity by directly recognizing and killing pathogens and activating T and B lymphocytes through cytokine release [282, 283]. γδ T cells kill cancer cells through direct recognition via TCRs and natural killer cell receptors (NKRs). They induce apoptosis using TRAIL, FAS ligand (FASL), and the granule exocytosis pathway, releasing perforin and granzymes. γδ T cells also mediate ADCC when tumor-specific antibodies are present. They enhance antitumor immunity by producing IFNγ and acting as antigen-presenting cells to activate αβ T cells. Additionally, they express the 4-1BB ligand (4-1BBL) to stimulate NK cells and induce antibody class switching in B cells. γδ T cells produce granulocyte–macrophage colony-stimulating factor (GM-CSF) to regulate dendritic cell (DC) infiltration. Their antitumor activity is further enhanced by IL-2, IL-15, IL-18, and IL-21 [284, 285] (Fig. 4). In MM, γδ T cells are activated by non-peptide antigens and stress-induced ligands, exhibiting cytotoxic activity by killing MM cells via perforin, granzyme, and death receptor pathways, and recognizing stress-induced ligands such as MICA/B and ULBP1-4 via the NKG2D receptor [286–289]. Additionally, they produce pro-inflammatory cytokines like IFN-γ and TNF-α, enhancing the immune response, and modulate the tumor microenvironment to promote anti-tumor immunity [285]. Therapeutically, γδ T cells can be expanded ex vivo for adoptive cell therapy and combined with monoclonal antibodies, checkpoint inhibitors, or chemotherapy to boost anti-tumor effects [290]. Challenges include achieving sufficient ex vivo expansion, overcoming the immunosuppressive microenvironment, and ensuring safety [291]. Preclinical and clinical trials are exploring the efficacy and safety of γδ T cell-based therapies in MM [55, 56, 292]. In hematological malignancies, Wilhelm et al. reported the infusion of allogeneic γδ T cells from healthy donors in patients with advanced refractory MM who were not eligible for allogeneic transplantation [57]. While CAR-γδ T cells showed promise, their limited proliferation and diversity led researchers to develop αβ T cells expressing γδ TCRs, known as TEGs [291]. These TEGs can target various hematological tumors, exhibiting potent antitumor activity, strong proliferation, and preserved CD4^+^ and CD8^+^ effector functions, leading to tumor eradication in the leukemia patient derived xenograft (PDX) model [293]. A phase I clinical trial (NCT04688853) is currently testing TEG002, an autologous T cell transduced with a specific γδ TCR, in patients with RRMM. In general, these cells hold significant potential as a therapeutic option, with further research needed to realize their full potential in improving patient outcomes.

#### Dendritic cell (DC) vaccination

**Fig. 5:**
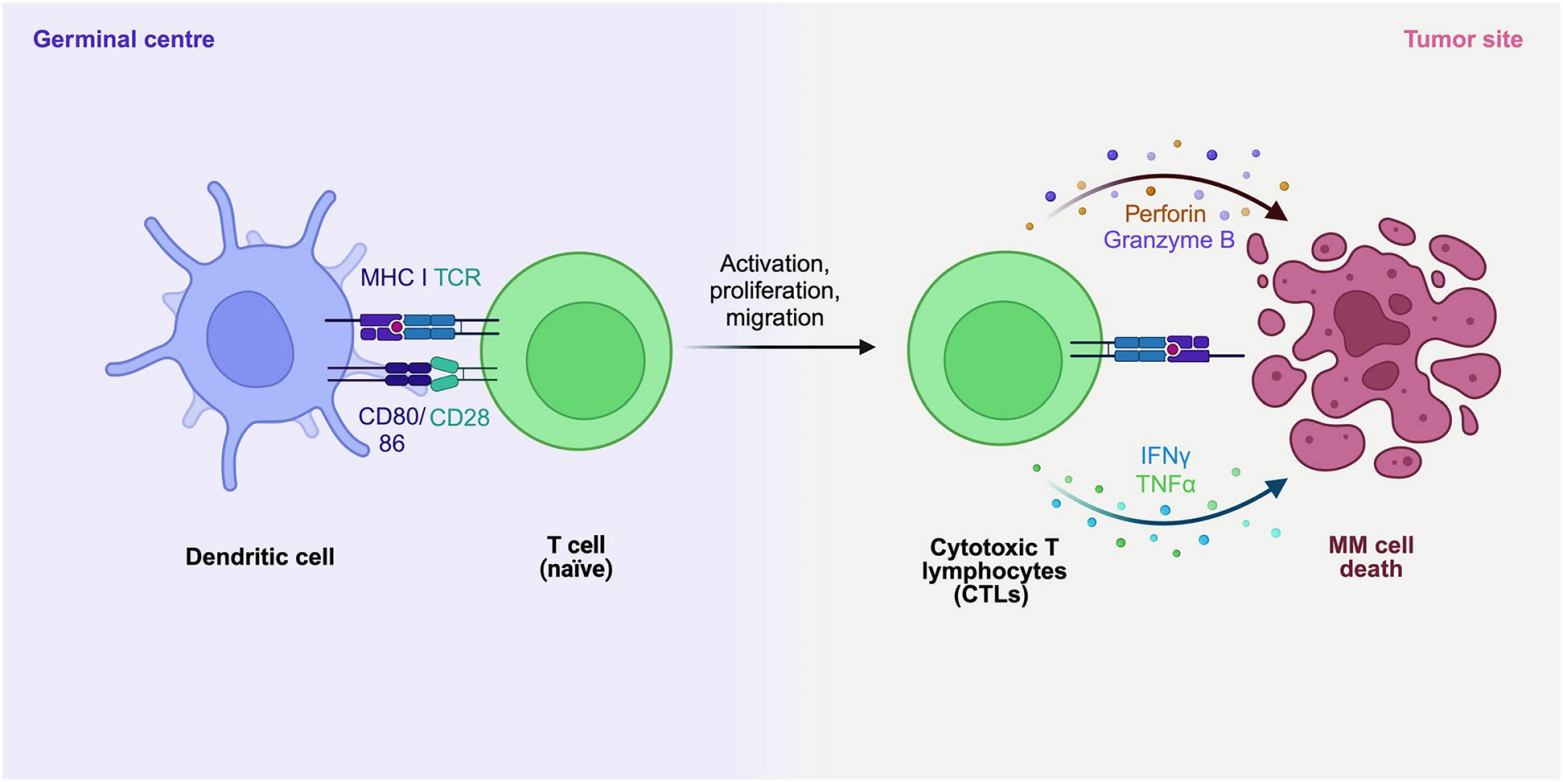
Immune activation of DCs in MM. The process begins with isolating DCs from the patient's blood via leukapheresis. These cells are then cultured with specific growth factors to differentiate into immature DCs, which are subsequently matured with stimuli like TNF-α. The mature DCs are loaded with myeloma-specific antigens from sources such as tumor lysates, peptides, or mRNA/DNA encoding myeloma antigens. Once loaded, these antigen-presenting DCs are injected back into the patient, typically intradermally or subcutaneously. The DCs then migrate to germinal centers (like lymph nodes), where they activate naïve T cells, leading to the generation of cytotoxic T lymphocytes (CTLs) that specifically target and kill MM cells. Additionally, helper T cells support the immune response by secreting cytokines. Some activated T cells become memory T cells, offering long-term surveillance against MM recurrence. Figure created with BioRender.com

Dendritic cell (DC) vaccines work by inducing and supporting an immune response to eradicate tumor cells. Autologous DCs, when pulsed with peptides or proteins derived from tumor lysates, can stimulate the production of cytotoxic T cells in MM patients [294–296] (Fig. 5). There are four main methods for using DCs as cell-based vaccines against cancer: co-culturing DCs with isolated autologous tumor tissues, co-culturing DCs with synthetic peptides or recombinant proteins of a tumor antigen, transfecting DCs with a specific plasmid to express tumor antigens, and fusing DCs with complete tumor cells using polyethylene glycol [297–299]. These methods enhance the ability of DC vaccines to stimulate a targeted immune response against MM. Han et al. showed that lentiviral-induced overexpression of calnexin (CNX) in DCs of MM patients enhanced MM-specific CD4 and CD8 T-cell responses, overcoming immune suppression [300]. CNX overexpression did not impact regulatory T cell (Treg) expansion. This suggests that improving antigen processing in DCs can lower the activation threshold of immune effector cells, potentially bypassing Treg-mediated suppression. Currently, the phase I clinical trial (NCT06435910) for this study is also ongoing. Genetically engineering DCs may thus enhance cancer immunotherapy. A randomized phase II trial (NCT02728102) found that combining DC/MM fusion vaccination with lenalidomide did not significantly increase CR rates one year post-transplant [58]. However, it did lead to a notable rise in circulating MM-reactive lymphocytes, suggesting enhanced tumor-specific immunity.

#### Cytokine-induced killer (CIK) cells

Cytokine-Induced Killer (CIK) cells are a diverse group of effector cells derived from PBMCs and expanded in vitro using IFN-γ, anti-CD3 antibody, and IL-2 [301, 302]. First described over 30 years ago, CIK cells are an innovative cancer immunotherapy strategy. They involve modifying and utilizing autologous or allogeneic CD3^+^CD56^−^ T cells and CD3^+^CD56^+^ NK-T cells, which can recognize tumor cells without HLA restriction [303, 304].

**Fig. 6:**
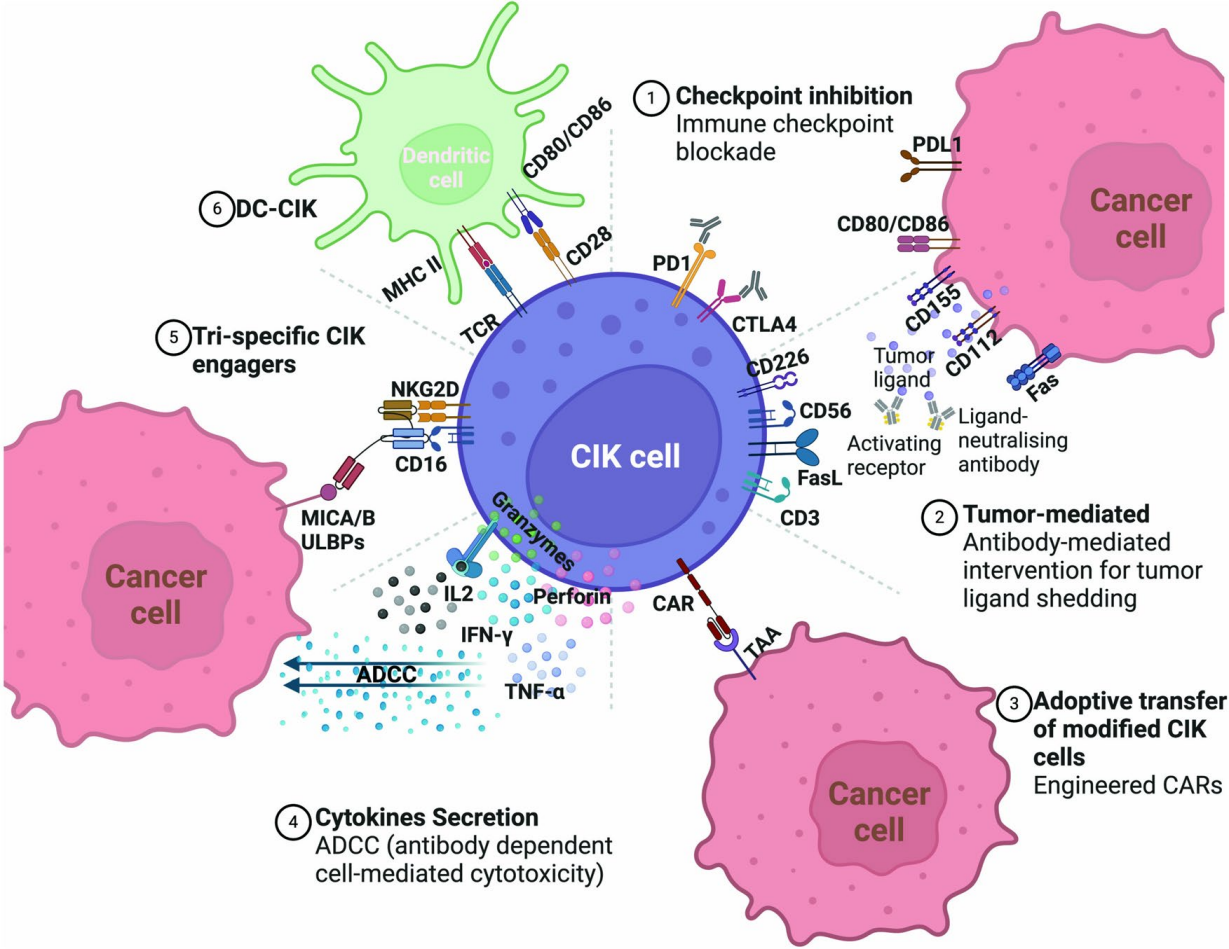
Approaches for CIK cell immunotherapy. CIK therapy employs various mechanisms to enhance its efficacy, such as combining with immune checkpoint inhibitors or epigenetic inhibitors, using antibody-mediated intervention to address tumor ligand shedding, and adopting the transfer of CIK cells engineered with CARs. It also includes ADCC, the use of tri-specific CIK engagers, and DC-CIK combinations. These approaches collectively improve the therapeutic potential of CIK cells in targeting and eliminating cancer cells. Figure created with BioRender.com

CIK cells possess potent antitumor activity due to their combined T cell (CD3^+^) and NK cell (CD56^+^) characteristics [305, 306]. They can be used in various therapeutic approaches (Fig. 6), including: combining with immune checkpoint inhibitors, antibody-mediated interventions to counter tumor ligand shedding, adoptive transfer of CIK cells engineered with CARs, ADCC, tri-specific CIK engagers, dendritic cell-CIK combinations (DC-CIK) and epigenetic inhibitors [307–313]. These mechanisms enable CIK cells to target MM cells through direct cytotoxicity and cytokine release. In the preclinical phase, Pu et al. demonstrated that combining HDAC inhibitors (HDACis) with CIK cells significantly enhances cytotoxicity against MM. This combination shows potential as a promising treatment option for MM patients. Additionally, Poles et al. showed that BCMA-CARs or affinity-optimized CD38-CARs with CIK cells not only spared normal hematopoietic cells but also exhibited a Th1-like cytokine profile, further supporting their therapeutic utility in MM [314].

Clinical trials in China have demonstrated that DC/CIK cells are safe and can induce clinical responses in MM patients, both as a standalone therapy and in combination with chemotherapy and other immunotherapies [315]. However, other clinical trials (NCT00477035, NCT00185757, NCT00460694) in the world have been completed without relevant clinical effect evaluations being reported. CIK cell therapy is notable for its broad antitumor activity, low risk of graft-versus-host disease (GVHD), and ease of expansion in vitro. Interest is growing in understanding the role of CIK cell therapy within the current and future landscape of immuno-oncology [316]. Ongoing research focuses on optimizing expansion protocols, exploring combination therapies, and developing personalized treatments. CIK cells present a promising immunotherapeutic approach for MM, with further research needed to solidify their role in clinical practice.

## Conclusion and future perspectives

This manuscript reviews the evolution of cell therapy for MM, highlighting recent advancements and future perspectives. Cell therapies have emerged as transformative options in MM treatment, demonstrating significant promise, particularly for patients with refractory or relapsed disease. Recent preclinical and clinical studies have underscored the efficacy of CAR-T cells, NK cells, and other immune effector cells. However, notable challenges persist in ensuring the safety and efficacy of these therapies, including CRS, neurotoxicity, and antigen escape, which complicate clinical outcomes.

Further investigation is essential to assess the durability of responses and the long-term safety profiles of these therapies. Each ACT approach for MM offers distinct strengths and limitations. CAR-T therapy, particularly targeting BCMA, currently demonstrates the highest efficacy and durability. BiTEs show significant promise in terms of accessibility and safety, while TCR therapy, NK cell therapy and other therapies are still in exploratory stages but may contribute to a more personalized treatment landscape in the future.

Off-the-shelf CAR-T and NK cell therapies are emerging as promising options for MM, offering advantages over traditional approaches. Their pre-manufactured nature allows for immediate availability, reduced costs, and consistent quality. Initial clinical trials targeting antigens like BCMA have shown high response rates in patients with RRMM. Nevertheless, challenges such as GVHD and antigen escape necessitate careful monitoring and innovative strategies.

Ongoing research aims to enhance the efficacy of these therapies through combination strategies and the identification of new therapeutic targets. Optimizing CAR-T cell design and delivery to minimize adverse effects and enhance persistence is critical. Developing next-generation CAR constructs, dual-targeting CARs, and safety switches is essential. Moreover, integrating cell therapy with other therapeutic modalities—such as immunomodulators, proteasome inhibitors, and monoclonal antibodies—could yield synergistic effects and address resistance mechanisms.

Exploring alternative immune effector cells, such as CAR-NK cells and TCR-engineered T cells, presents further avenues for effective treatment. Establishing robust biomarkers for patient selection and response monitoring is vital for personalizing treatment strategies.

Adoptive cellular immunotherapy has opened new therapeutic avenues for patients with MM, especially those with limited options. While CAR-T cell therapies demonstrate transformative potential, challenges such as manufacturing complexity, toxicities, and immune evasion remain. Emerging strategies, including NK cell therapies, cytokine-induced killer (CIK) cell therapies, and bispecific antibodies, hold promise for overcoming the limitations of existing therapies. Future research should prioritize optimizing these strategies, reducing associated toxicities, and exploring novel targets to achieve sustained and widespread responses. Collaborative efforts among clinicians, researchers, and industry stakeholders will be pivotal in translating these advances from bench to bedside, ultimately leading to more effective and durable treatments for patients with MM.

## Supplementary Information

Supplementary Material 1.

## Data Availability

No datasets were generated or analysed during the current study.

## Abbreviations

MM: Multiple myeloma
ACT: Adoptive cellular immunotherapy
ASCT: Autologous stem cell transplantation
CAR: Chimeric antigen receptor
BCMA: B-cell maturation antigen
NK: Natural killer
TCR: T-cell receptor
MGUS: Monoclonal gammopathy of undetermined significance
TME: Tumor microenvironment
RRMM: Relapsed/refractory multiple myeloma
FDA: The United States Food and Drug Administration
DCs: Dendritic cells
ATILs: Adoptive transfer of tumor-infiltrating lymphocytes
BiCEs: Bispecific immune cell engagers
TriCEs: Trispecific immune cell engagers
LAK: Lymphokine-activated killer
TILs: Tumor-infiltrating lymphocytes
PBMCs: Peripheral blood mononuclear cells
HDM: High-dose melphalan
PFS: Progression-free
mPFS: Median progression-free
OS: Overall survival
mOS: Median overall survival
VTd: Bortezomib, thalidomide, and dexamethasone
VRd: Bortezomib, lenalidomide, and dexamethasone
D-VTd: VTd combined with daratumumab
D-VRd: VRd combined with daratumumab
I-VRd: VRd combined with isatuximab
ASCO: American Society of Clinical Oncology
ESMO: European Society for Medical Oncology
EBMT: European Bone Marrow Transplantation
PI: Proteasome inhibitor
IMiD: Immunomodulatory drug
NDMM: Newly diagnosed multiple myeloma
CR: Complete response
sCR: Stringent complete response
MRD: Minimal residual disease
KRd: Carfilzomib, lenalidomide and dexamethasone
D-KRd: KRd combined with daratumumab
I-KRd: KRd combined with isatuximab
ADCC: Antibody-dependent cellular cytotoxicity
HSC: Hematopoietic stem cells
G-CSF: Granulocyte colony-stimulating factor
MHC: Major histocompatibility complex
pMHC: Peptide-MHC
ITAMs: Immunoreceptor tyrosine-based activation motifs
CTA: Cancer/testis antigen
MAGE: Melanoma antigen gene
scFv: Single-chain variable fragment
APRIL: A proliferation-inducing ligand
BAFF: B-cell activating factor
CRS: Cytokine release syndrome
ICANS: Immune effector cell-associated neurotoxicity syndrome
ORR: Overall response rate
VGPR: Very good partial response
CTX: Cyclophosphamide
FAM: Fludarabine
pt: Per test
mDOR: Median duration of response
DOR: Duration of response
DLTs: Dose-limiting toxicities
SLAMF7: Signaling lymphocytic activation molecule family member 7
ICANS: Immune effector cell-associated neurotoxicity syndrome
GPRC5D: G protein-coupled receptor class C group 5 member D
PIs: Proteasome inhibitors
FcRH5: Fc receptor-homolog 5
TCEs: T-cell engagers
TRUCKS: T cells redirected for universal cytokine-mediated killing
GVHD: Graft-versus-host disease
HLAs: Human leukocyte antigens
iPSCs: Induced pluripotent stem cells
sBCMA: Soluble BCMA
BsAb: Bispecific antibody
TsAbs: Trispecific antibodies
NKCEs: NK cell engagers
TandAb: Tandem diabody
TriKEs): Trifunctional NK cell engagers
IL-2: Interleukin-2
rIL-2: Recombinant interleukin-2
ABMT: Autologous bone marrow transplantation
IFNγ: Interferon-γ
NKRs: Natural killer cell receptors
FASL: FAS ligand
4-1BBL: 4-1BB ligand
GM-CSF: Granulocyte–macrophage colony-stimulating factor
PDX: Patient derived xenograft
CNX: Calnexin
Tregs: Regulatory T cells
CTLs: Cytotoxic T lymphocytes
CIK: Cytokine-Induced killer
DC-CIK: Dendritic cell-CIK combinations
HDACis: Histone deacetylase inhibitors
BMMCs: Bone marrow mononuclear cells
MM Id-Ig: Multiple myeloma-specific idiotype immunoglobulins
