# Supplementary material for: Advances in adoptive cellular immunotherapy and therapeutic breakthroughs in multiple myeloma
Source: Exp Hematol Oncol. 2024 Oct 28;13:105. doi: 10.1186/s40164-024-00576-6 (PMC11514856; doi:10.1186/s40164-024-00576-6)
Supplement: Supplementary file 1 — Supplementary Material 1. [file 40164_2024_576_MOESM1_ESM.docx]

**Supplementary** **table 1** Preclinical studies of adoptive cell therapies for MM treatment.

| **Type** | **Experimental model** | **Conclusions** | **References** |
| --- | --- | --- | --- |
| TCR gene engineered T cells | **Design**: T cells transduced with a clinical-grade HLA-A2-restricted NY-ESO-1157-165-specific TCR.  **Test**: in vivo- Immunodeficient NSG mice inoculated with U266 cell line. | NY-ESO-1 TCR single edited stem and central memory T cells to treat MM without GVHD. | [97] |
| CAR-T cells | **Design**: Four second- generation humanized BCMA-directed CAR constructs: Anti-BCMA-scFv-CD8-41BB-CD3ζ (H8BB), Anti-BCMA-scFv-CD8-CD28-CD3ζ (H828), Anti-BCMA-scFv-IgG4-CD28-41BB-CD3ζ (ICBB) and Anti-BCMA-scFv-IgG4-CD28-CD28-CD3ζ (IC28).  **Test**: in vitro- NCI-H929 and RPMI-8226 myeloma cell lines.  in vivo- Immunodeficient NSG mice inoculated with NCI-H929 cell line. | Anti-BCMA-scFv-CD8-41BB-CD3ζ (H8BB) displayed superior anti-tumoral activity both in vitro and long-term in vivo efficacy. | [130] |
|  | **Design**: Descartes-08 (a novel CD8^+^ CAR-T cell product) and Anti-BCMA-scFv-CD8-CD28-CD3ζ.  **Test**: in vitro- BMMCs or CD138^+^ cells from MM patients, NCI-H929 and U266 myeloma cell lines.  in vivo- Immunodeficient NSG mice inoculated with MM1S-luc cells. | CD8^+^ anti-BCMA mRNA CAR-T cells demonstrate high levels of degranulation, cytokine secretion, proliferation, and cytotoxicity against MM cell lines and patient MM cells, regardless of drug resistance and in a specific, dose- and time-dependent manner. | [131] |
|  | **Design**: Anti-BCMA(J22.9)-scFv-CD8-41BB-CD3ζ.  **Test**: in vitro- ARP1 and U266 myeloma cell lines.  in vivo- Immunodeficient NSG mice inoculated with ARP1 cells. | This study optimized the humanized CAR targeting the mature antigen of myeloma B cells, demonstrating the potential of CAR-T cells in the treatment of MM. | [132] |
|  | **Design**: Allogeneic Anti-BCMA-scFv-CD8-41BB-CD3ζ.  **Test**: in vitro- MM.1S myeloma cell line.  in vivo- Immunodeficient NSG mice inoculated with MM.1S cells. | This novel off-the-shelf allogeneic BCMA CAR-T product is a promising candidate for clinical evaluation. | [133] |
|  | **Design**: Allogeneic Anti-BCMA-scFv-CD8-41BB-CD3ζ and BCMA TurboCAR-T cells.  **Test**: in vitro- MM.1S myeloma cell line.  in vivo- Immunodeficient NSG mice inoculated with MM.1S cells. | The study showed that the potential of constitutive Turbodomains to achieve selective potentiation of CAR-T cells and demonstrate the safety and efficacy of allogeneic BCMA TurboCAR-T cells, supporting clinical evaluation in MM. | [134] |
|  | **Design**: BCMA-directed scFv-based C11D5.3 CAR.  **Test**: in vitro- RPMI8226, NCI-H929, U266 and MM.1S myeloma cell line.  in vivo- Immunodeficient NSG mice inoculated with U266 or MM.1S cells. | These efficacy data supported the evaluation of CAR-T-ddBCMA cells for the treatment of RRMM and has led to the advancement of a CAR-T-ddBCMA cell candidate into phase I clinical trials. | [135] |
|  | **Design**: Anti-BCMA-scFv-CD19-scFv-CD8-41BB-CD3ζ (tan-CAR).  **Test**: in vitro- RPMI8226 myeloma cell line.  in vivo- Immunodeficient NSG mice inoculated with K562 cells. | The study introduces a novel tan-CAR-T cell design that effectively targets both CD19 and BCMA antigen-positive tumor cells, showing significant anti-tumor efficacy in vitro and in vivo. This strategy could potentially lower relapse rates associated with single scFv-CAR-T cell treatments. | [156] |
|  | **Design**: Anti-CD38-scFv-CD8-41BB-CD3ζ.  **Test**: in vitro- RPMI8226 and KMM1 myeloma cell lines, primary MM cells from myeloma patients. | The study showed that human T cells with the anti-CD38-CAR were highly cytotoxic against myeloma cells strongly expressing CD38. Improvements to immunotherapy using autologous T cells transduced with the anti-CD38-CAR might shed further light on the treatment of MM patients with a poor prognosis. | [165] |
|  | **Design**: Anti-CD38-scFv-CD8-41BB-CD3ζ.  **Test**: in vitro- U8226 and UM9 myeloma cell lines, primary MM cells from patients.  In vivo- RAG2γc mice inoculated with UM9 cells. | These results signify the potential importance of anti-CD38-CAR-T cells as therapeutic tools for CD38^+^ malignancies and warrant further efforts to diminish the undesired effects of this immunotherapy using appropriate strategies in MM. | [166] |
|  | **Design**: Anti-CD38-scFv-CD8-41BB-CD3ζ.  **Test**: in vitro- LP-1, RPMI 8226, OPM-2, and MOLP8 myeloma cell lines, primary MM cells from myeloma patients.  In vivo- Immunodeficient NSG mice inoculated with RPMI8226 cells. | These results demonstrate that the CD38-CAR-T cells constructed with the anti-CD38 nanobody are a promising approach for the treatment of MM. | [167] |
|  | **Design**: Anti-CD38-scFv-CD8-41BB-CD3ζ.  **Test**: in vitro- KMS12, MM.1S , RPMI 8226 and U266 myeloma cell lines.  In vivo- Immunodeficient NSG mice inoculated with RPMI8226 cells. | Deploying CAR-T 38 against multiple CD38-expressing malignancies is significant because it expands the potential for this novel therapy to affect diverse patient populations. | [168] |
|  | **Design**: SLAMF7-targeted CAR-T cell.  **Test**: in vitro- MM.1S, NCI-H929 and OPM-2 myeloma cell lines.  In vivo- Immunodeficient NSG mice inoculated with MM.1S cells. | The data illustrated the potential use of SLAMF7-CAR-T cell therapy as an effective treatment against MM and provided novel insights into the consequences of targeting SLAMF7 for the normal lymphocyte compartment. | [177] |
|  | **Design**: Anti-SLAMF7-scFv-CD38-scFv-CD8-41BB-CD3ζ(DCAR).  **Test**: in vitro- MM.1S, NCI-H929 and KMS-11 myeloma cell lines.  In vivo- Immunodeficient NSG mice inoculated with MM.1S cells. | DCAR-T cells could provide a safe and efficient alternative to anti-BCMA CAR-T cell therapy to treat patients with MM. | [178] |
|  | **Design**: Anti-Luc90 CS1-scFv-CD28-41BB-CD3ζ.  **Test**: in vitro- MM.1S and OPM-2 myeloma cell lines.  In vivo- Immunodeficient NSG mice inoculated with MM.1S-CG cells. | The data demonstrated targeting the distal V domain of CS1 could be an effective CAR-T treatment for myeloma patients and deletion of CS1 in clinical production did not provide an added benefit using in vivo immunodeficient NSG preclinical models. | [179] |
|  | **Design**: Anti-BCMA-scFv-41BB-CD3ζ[2A]GPRC5D-scFv-41BB-CD3ζ.  **Test**: in vitro- MM.1S, RPMI8226, OPM-2 and NIH-3T3 myeloma cell lines.  In vivo- Immunodeficient NSG mice inoculated with OPM-2 cells. | The data suggested that dual-targeted CAR-T cell therapy targeting both BCMA and GPRC5D could prevent MM relapse caused by BCMA escape | [185] |
|  | **Design**: Anti-CD138-scFv-CD28- CD3ζ.  **Test**: in vitro- OPM-2, RPMI8226, U266-B1 and MMS1 myeloma cell lines.  In vivo- Immunodeficient NSG mice inoculated with OPM-2 cells. | Preclinical studies confirmed that CD138-specific CAR-T cells, when used adoptively, show no cytotoxicity towards normal epithelial or endothelial cells. This supports their potential use in treating RRMM. | [197] |
|  | **Design**: Anti-FcRH5-scFv-CD8-CD28-CD3ζ.  **Test**: in vitro- NCI-H929 and MM.1S myeloma cell lines.  In vivo- Immunodeficient NSG mice inoculated with NCI-H929 cells. | CAR-T cells targeting FcRH5 provide robust tumour-specific responses in murine xenograft models of MM. | [201] |
| CAR-NK cells | **Design**: Anti-BCMA-scFv-CD8-CD28-CD3ζ-IL15.  **Test**: in vitro- NCI-H929 and MM.1S myeloma cell lines.  In vivo- Immunodeficient NSG mice inoculated with MM.1S cells. | These results showed that the single VHH-directed BCMA CAR-NK cells exhibited remarkable specific killing ability, making them a potential candidate for immunotherapy in MM treatment. | [233] |
|  | **Design**: BCMA/GPRC5D dual-targeted CAR-NK.  **Test**: In vivo- Immunodeficient NSG mice inoculated with a human MM cell line (Luc+). | These results indicated that BCMA/GPRC5D dual-targeted CAR-NK cell therapy has great potential to be used in MM patients as an off-the-shelf therapeutic product to further improve response duration compared to BCMA or GPRC5D single targeted cell therapy. | [234] |
|  | **Design**: CD138 targeted CAR-NK.  **Test**: in vitro- RPMI8226, U266 and NCI-H929 myeloma cell lines.  In vivo- Immunodeficient NSG mice inoculated with 5 × 10^6^ RPMI8226 cells. | CD138-targeted CAR-NK cells target CD138-positive plasmacytic malignancies may enhance remission quality and extend remission duration in MM patients post-chemotherapy. | [235] |
|  | **Design**: SLAMF7 targeted CAR-NK.  **Test**: in vitro- L363, U266 and IM9 cell lines.  In vivo- Immunodeficient NSG mice inoculated with 5 × 10^5^ lIM9 cells. | SLAMF7-targeted CAR-NK cell therapy could be a promising treatment for MM. | [236] |
|  | **Design**: Anti-NKG2D-4-1BB-CD3z-CAR.  **Test**: in vitro- MM.1S , MM.1R, U-266, L-363, OPM-2, NCI-H929, JJN-3 , RPMI-8826 and SK-MM-2 myeloma cells.  In vivo- Immunodeficient NSG mice inoculated with U266 cells. | These findings showed that autologous activated and expanded NK cells from MM patients could be safely engineered to express NKG2D-CAR, enhancing their anti-myeloma activity. This supported the potential of NKG2D-CAR-NK cell therapy as an effective treatment for MM. | [237] |
|  | **Design**: CD38/GPRC5D dual-targeted CAR-NK.  **Test**: In vivo- Immunodeficient NSG mice inoculated with MM.1S cells. | FT555 is a CAR-NK cell derived from a clonal iPSC line, engineered to target both GPRC5D and CD38 with daratumumab. This standardized, scalable platform provides effective off-the-shelf therapy for MM. | [238] |
| LAK cells | **Design**: Comparison of the killing effect of LAK cells induced by rIL-2 in the peripheral blood of healthy donors and MM patients.  **Test**: Malignant plasma cells. | These results suggested that the in vivo administration of IL2 in MM deserves further evaluation, particularly for its potential to control minimal residual disease. | [271] |
| γδ T cells | **Design**: bsAb CD40-Vγ9Vδ2 T cell engager (LAVA-1278).  **Test**: in vitro- MM.1S myeloma cells.  In vivo- Immunodeficient NSG mice inoculated with MM.1S cells. | It may represent a potential candidate for the development of novel treatments for B-cell malignancies (including MM). | [289] |
| Dendritic cells | **Design**: Supraphysiological expression of calnexin (CNX) using lentiviral (LV) vectors in DCs of MM patients.  **Test**: DCs from MM patients. | MM cell lysates or MM Id-Ig specifically induced the expansion of peripheral CD4(+)CD25(high)FoxP3(high) Tregs in vitro. | [298] |
| CIK cells | **Design**: CIK cells from PBMCs (healthy donors) combined with HDACis.  **Test**: in vitro- OPM-2, U266, NCI-H929 and RPMI8226 myeloma cells. | NKG2D/NKG2D-ligand interactions activating NK/NKT cells may contribute to enhanced myeloma cell lysis in response to HDACis treatment by CIK cells. | [312] |
|  | **Design**: CD38 targeted CAR-CIK and BCMA targeted CAR-CIK cells.  **Test**: in vitro- UM9, MM1.s and MM1.s-CD1d myeloma cells. | iNKT cells expressing either BCMA-CARs or affinity-optimized CD38-CARs spared normal hematopoietic cells and displayed a Th1-like cytokine profile, indicating their therapeutic utility in MM. | [313] |

MM: multiple myeloma; RRMM: relapsed/refractory multiple myeloma; GVDH: graft-versus-host disease; BMMCs: bone marrow mononuclear cells; MM Id-Ig: multiple myeloma-specific idiotype immunoglobulins; Tregs: T regulatory cells; HDACis: histone deacetylase inhibitors.
